# Palmitic Acid Promotes Antiviral Innate Immunity via ZDHHC20‐Mediated CMPK2 Palmitoylation

**DOI:** 10.1002/advs.75209

**Published:** 2026-04-21

**Authors:** Yujia Wang, Zenghui Cui, Yunkai Zhang, Zhiqing Li, Xuetao Cao

**Affiliations:** ^1^ Department of Immunology, Center for Immunotherapy, Institute of Basic Medical Sciences & School of Basic Medicine Chinese Academy of Medical Sciences & Peking Union Medical College Beijing China; ^2^ Institute of Immunology, College of Life Sciences Nankai University Tianjin China; ^3^ National Key Laboratory of Immunity and Inflammation, Institute of Immunology Naval Medical University Shanghai China

**Keywords:** CMPK2, IFN‐I, immunometabolism, innate immune response, palmitoylation

## Abstract

While immunometabolic crosstalk is critical for antiviral defence, the regulation of this process, particularly through post‐translational modifications, remains incompletely understood. How specific metabolites and associated modifications orchestrate antiviral immunity remains unclear. By screening a metabolic chemical library, we identify palmitic acid (PA) as an activator of antiviral immunity in macrophages. PA induces UMP‐CMP kinase 2 (CMPK2) palmitoylation, maintaining its mitochondrial localization. CMPK2 is vital for the production of 3'‐deoxy‐3',4',‐didehydrocytidine triphosphate (ddhCTP) and the stabilization of mitochondrial antiviral signaling protein (MAVS), both of which are crucial for defence against RNA viruses. Cmpk2 deficiency impairs IFN‐I production and increases viral replication. Furthermore, the palmitoyl transferase ZDHHC20 catalyzes CMPK2 palmitoylation at cysteines 137 and 153, which are depalmitoylated by the thioesterase PPT1. PPT1 deficiency restores CMPK2 palmitoylation and antiviral immunity. Both a palm oil‐rich diet and the in vivo administration of the PPT1 inhibitor DC661 increase IFN‐I production. Therefore, the PA‐ZDHHC20‐CMPK2‐PPT1 axis enhances the antiviral response, indicating that targeting PPT1 has the potential to treat RNA virus infections.

## Graphical abstract

Metabolites have important functions in innate immune activation and regulation. Wang et al. uncover metabolic regulation of antiviral immunity through CMPK2 palmitoylation, which regulates CMPK2 mitochondrial localization and is promoted by ZDHHC20 but reversed by PPT1, inhibition of which antagonizes viral infection in mice.

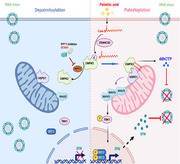

## 1 Introduction

The innate immune response provides the first line of host defence against invading pathogens. As such, innate immunity is controlled by an elaborate regulatory network, and it is increasingly appreciated that metabolic pathways play important nonmetabolic roles in this process. Immunometabolism has emerged as a key focus for biomedical research since metabolic changes in immune cells can critically affect immune cell activation and immune effector responses [1]. Recent work has linked metabolites such as glutathione, [2] manganese, [3] serine, [4] adenine, [5] and itaconate [6] to innate immune activation and regulation. In this context, exploring the function of metabolites from distinct pathways could provide novel insights into immuno‐metabolic crosstalk and deepen the understanding of the pathogenesis of metabolic disorders.

Post‐translational modifications (PTMs), such as acetylation [7], ubiquitination [8], and glycosylation [9] of innate sensors and downstream signaling molecules, play significant roles in the activation or repression of the innate immune response. Several metabolites play well‐established roles in regulating distinct immunological processes through covalent modification, [10] for example, itaconate [11] and lactate [12]. S‐palmitoylation is the covalent attachment of long‐chain fatty acyl groups (typically saturated 16C palmitate) to proteins via a cysteine (Cys) residue through a thioester bond. Both mammalian and viral proteins are palmitoylated, influencing properties such as subcellular distribution, conformation, activity, stability, aggregation, and protein‐protein interactions. S‐palmitoylation is connected to the innate immune response to DNA viruses, such as STING [13] and cGAS [14]. However, the broader role of S‐palmitoylation in antiviral immunity remains unclear.

In this study, we screened a metabolism‐modulating chemical library to identify metabolites that regulate innate antiviral immunity. Here, we report that palmitic acid (PA) enhances antiviral innate immunity via palmitoylation. Mechanistically, CMPK2 is S‐palmitoylated on Cys137 and 153 by ZDHHC20 upon RNA virus infection. Palmitoylated CMPK2 catalyzes the production of ddhCTP, an antiviral factor that suppresses viral replication. However, CMPK2 has a second antiviral effect, where it interacts with mitochondrial HSPD1 to maintain mitochondrial homeostasis and promote the abundance of MAVS, which in turn enhances innate immunity. Thus, S‐palmitoylation anchors CMPK2 to mitochondria, which is vital for both ddhCTP production and the MAVS‐mediated interferon (IFN) pathway. In contrast, PPT1 depalmitoylates CMPK2, and its inhibitor DC661 enhances the antiviral response in vivo, suggesting a potential strategy for combatting viral infections.

## 2 Results

### 2.1 Palmitic Acid and ZDHHC20‐Mediated S‐Palmitoylation Enhances the Antiviral Innate Response

**FIGURE 1:**
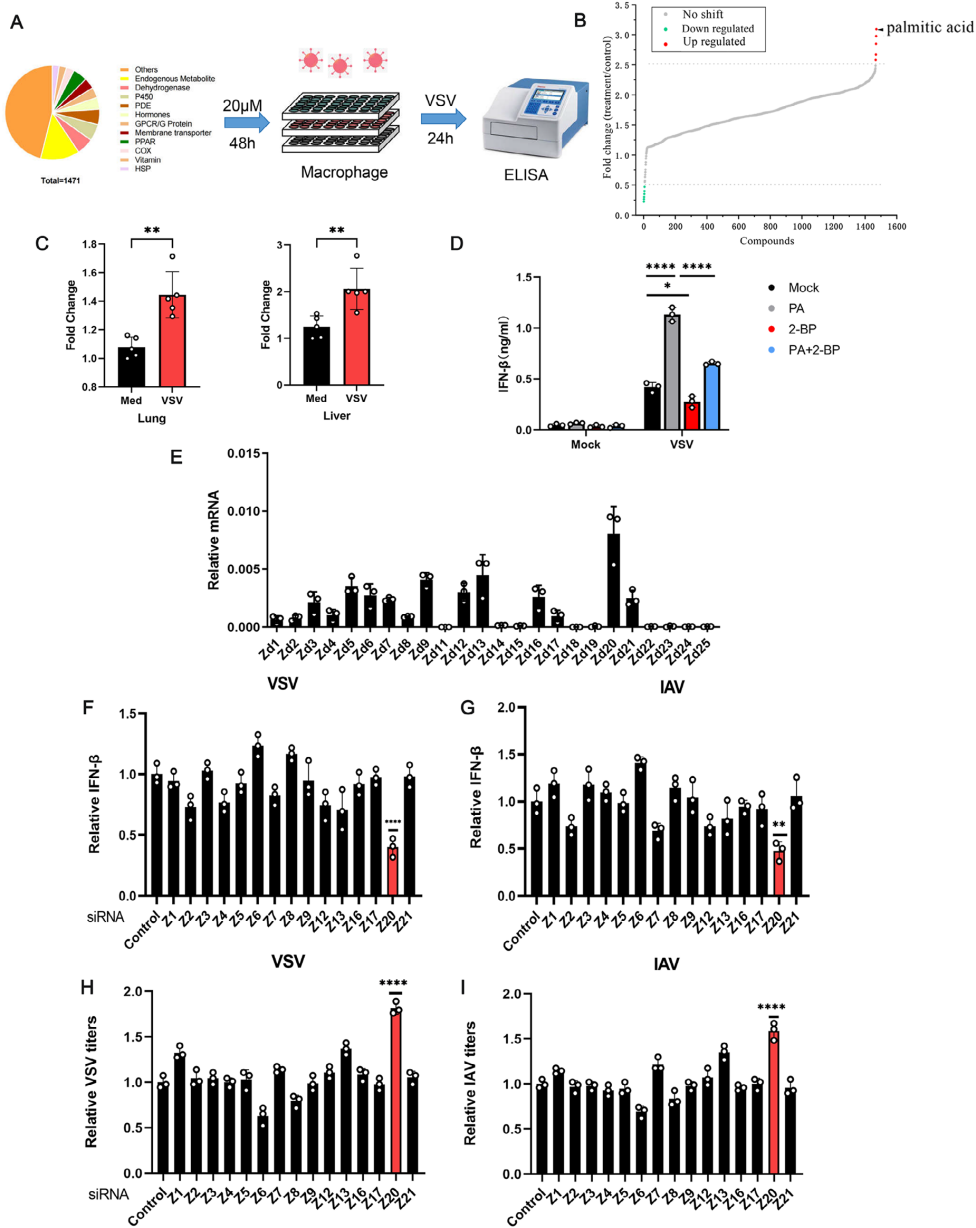
Palmitic acid (PA) enhances antiviral innate immunity via palmitoylation. (A) Schematic of our high‐throughput pharmacological screen. Peritoneal macrophages (PMs) were treated with compounds from a metabolism‐modulating chemical library at 20 µM for 48 h. IFN‐β in the supernatants was then detected by ELISA after 24 h of VSV challenge. (B) Relative IFN‐β levels secreted by PMs after treatment with individual compounds from the chemical library or DMSO and then infected with VSV for 24 h. The data were normalized to those of DMSO‐treated cells. (C) LC‐MS/MS analysis of PA in the organs of mice infected with VSV for 3 h. (D) ELISA of IFN‐β in the supernatants of BMDMs pretreated with/without PA (50 µM) or 2‐BP for 8 h and then stimulated with/without VSV for 12 h. (E) q‐PCR analysis of the expression of each ZDHHC enzyme in BMDMs. (F) ELISA of IFN‐β in the supernatants of BMDMs transfected with the indicated siRNAs and then infected with VSV. (G) ELISA of IFN‐β in the supernatants of BMDMs transfected with the indicated siRNAs and then infected with IAV. (H) Determination of the VSV load using the 50% tissue culture infectious dose (TCID50) assay in the supernatants of the cells described in (F). (I) Determination of the IAV load by a TCID50 assay in the supernatants of the cells described in (G). Statistical analysis was performed by unpaired two‐tailed Student's *t*‐test (C); one‐way ANOVA (D and F–I), ^*^ *p* < 0.05, ^**^ *p* < 0.01, ^****^ *p* < 0.0001.

We screened a metabolism‐modulating chemical library, including 1471 metabolites and inhibitors/agonists of metabolic enzymes, in macrophages using IFN‐β production as a readout (Figure 1A). This screen revealed palmitic acid as a positive regulator of IFN‐β production during Vesicular Stomatitis Virus (VSV) infection (Figure 1B). We observed an increase of PA in both lungs and livers of mice in the early stage of VSV infection, indicating a potential role for PA in priming the antiviral immune response (Figure 1C). FASN (fatty acid synthase) is the rate‐limiting enzyme to generate cellular palmitic acid. [15] Both inhibitor and siRNA targeting FASN dampened IFN‐I production and promoted virus replication (Figure S1), so we speculated that endogenous PA actively regulated host response. PA‐induced stimulation of IFN‐I was efficiently antagonized by the nonspecific palmitoyl‐transferase (ZDHHC) inhibitor 2‐Bromopalmitic acid (2‐BP) [16] (Figure 1D), indicating that the regulation of the antiviral immune response by PA was dependent to some extent on its linkage on cysteines, that is, protein S‐palmitoylation. To identify the palmitoyl transferases that function in antiviral innate immunity in macrophages, we examined the expression levels of 24‐well‐established ZDHHCs in bone marrow‐derived macrophages (BMDMs) (Figure 1E). We subsequently conducted a small interfering RNA (siRNA) screen targeting ZDHHCs whose expression is relatively high in macrophages. We found that *Zdhhc20* knockdown significantly increased viral titer and suppressed IFN‐β production upon infection with either VSV or Influenza A virus (IAV) (Figure 1F–I), suggesting that Zdhhc20 promotes the antiviral response in macrophages.

### 2.2 ZDHHC20 Deficiency Reduces IFN‐I Induction by RNA Viruses

**FIGURE 2:**
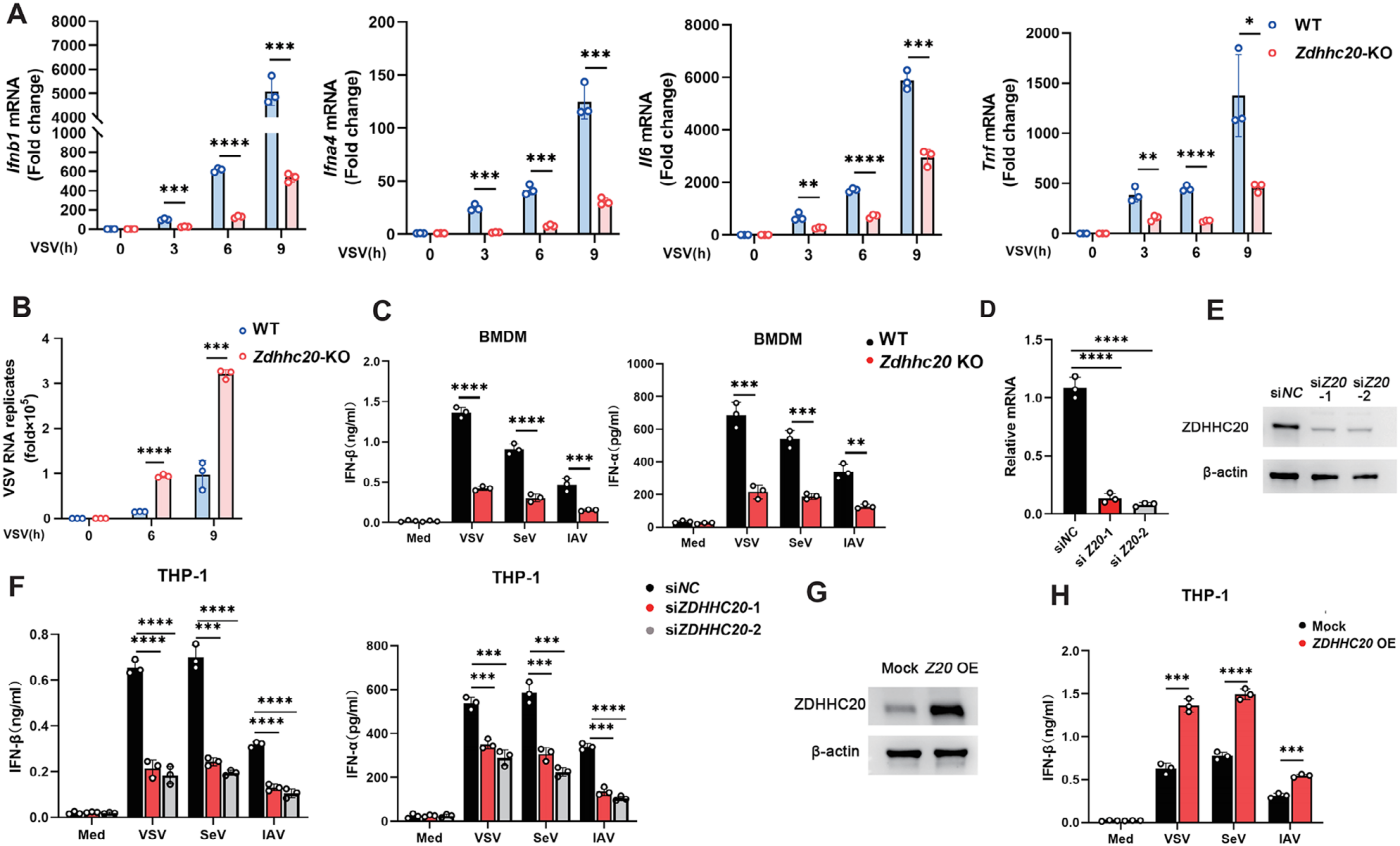
ZDHHC20 facilitates antiviral immune response in vitro. (A) q‐PCR analysis of *IFN‐β*, *IFN‐α*, *IL‐6*, and *TNF* mRNA expression in *Zdhhc20^+/+^* and *Zdhhc20^−/−^* BMDMs infected with VSV for the indicated durations. (B) q‐PCR analysis of VSV RNA in *Zdhhc20^+/+^* and *Zdhhc20^−/−^* BMDMs infected with VSV for the indicated durations. (C) ELISA of IFN‐β and IFN‐α in the supernatants of *Zdhhc20^+/+^* and *Zdhhc20^−/−^* BMDMs infected with VSV, SeV, or IAV for 12 h. (D) q‐PCR analysis of *ZDHHC20* expression in THP‐1 cells 48 h after transfection with control siRNA or two separate *ZDHHC20*‐specific siRNAs. (E) Immunoblot analysis of ZDHHC20 expression in THP‐1 cells treated as described in (D). (F) ELISA of IFN‐β and IFN‐α in the supernatants of THP‐1 cells treated as described in (D) and then infected with VSV, SeV, or IAV for 12 h. (G) Immunoblot analysis of *ZDHHC20* expression in THP‐1 cells transfected with mock or *ZDHHC20*‐OE lentivirus. (H) ELISA of IFN‐β in the supernatants of THP‐1 cells treated as described in (G) and then infected with VSV, SeV, or IAV for 12 h. Statistical analysis was performed by one‐way ANOVA (A–D, F, and H). ^*^ *p* < 0.05, ^**^ *p* < 0.01, ^***^ *p* < 0.001, ^****^ *p* < 0.0001.

To further elucidate the role of ZDHHC20 in antiviral innate immunity, we generated *Zdhhc20*‐deficient mice. Compared with their wild‐type (WT) littermates, *Zdhhc20* ^−/−^ mice showed no evident physiological or behavioral differences. Flow cytometry demonstrated that *Zdhhc20* deficiency did not influence the development of either innate or adaptive immune cells (Figure S2). To investigate the role of ZDHHC20 during viral infection, we challenged *Zdhhc20* ^+/+^ and *Zdhhc20* ^−/−^ macrophages with VSV. *Zdhhc20* depletion decreased the expression of IFN‐β, IFN‐α, and the proinflammatory cytokines IL‐6 and TNF in BMDMs (Figure 2A). Correspondingly, the replication of VSV was increased in *Zdhhc20* ^−/−^ BMDMs (Figure 2B). We observed similar results when BMDMs were infected with two other RNA viruses (Sendai virus (SeV) and IAV) (Figure 2C). Both gene silencing (Figure 2D–F) and overexpression (OE) (Figure 2G,H) experiments demonstrated that ZDHHC20 promoted IFN‐I production in human THP‐1 cells.

### 2.3 Myeloid‐Specific Zdhhc20 Deficiency Sensitizes Mice to Viral Infection with Reduced IFN‐I Production

**FIGURE 3:**
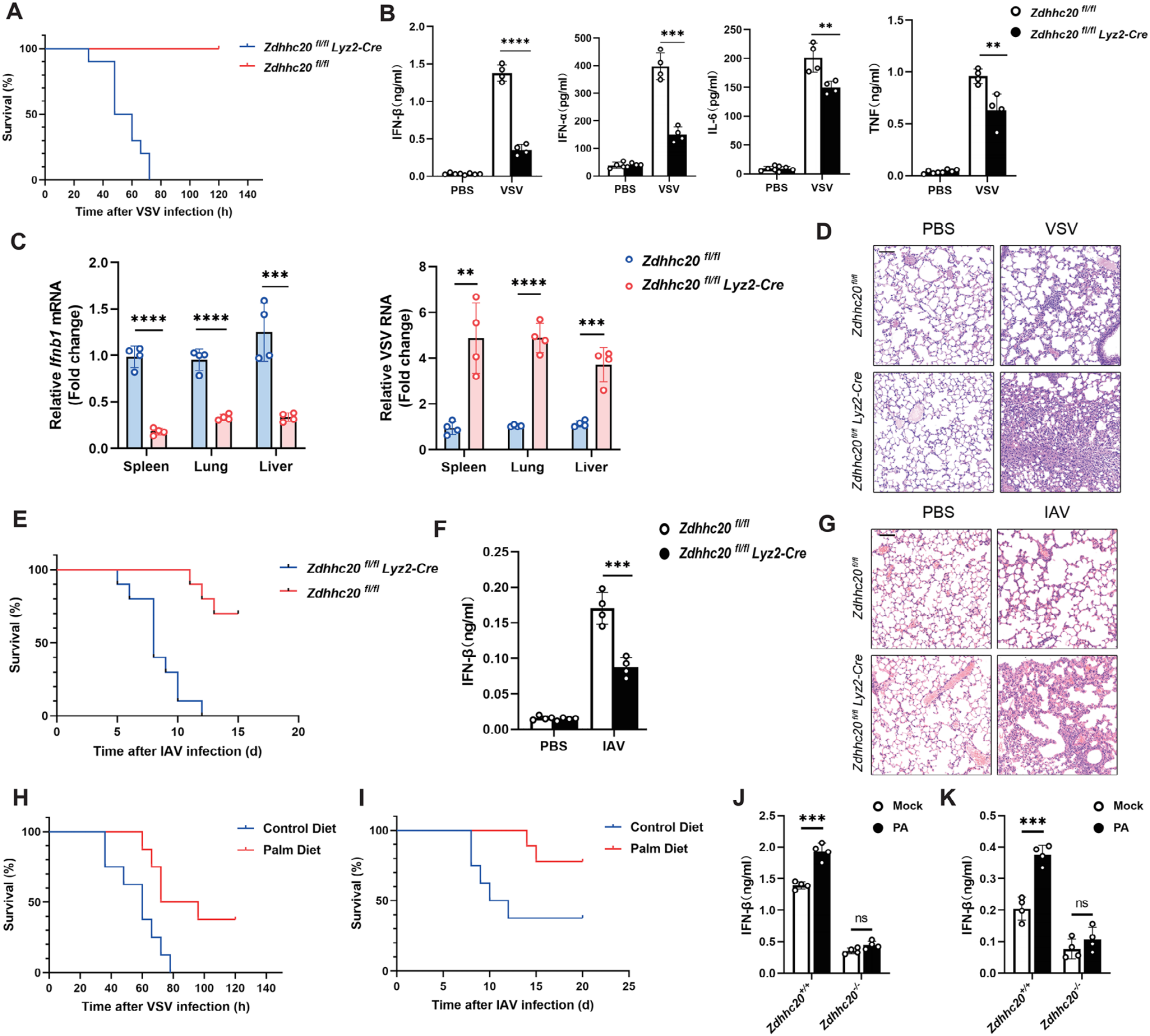
In vivo deficiency of *Zdhhc20* sensitizes mice to viral infection. (A) Survival data for 8‐week‐old *Zdhhc20* cKO and littermate mice (n=10) after intraperitoneal injection of VSV (1 × 10^7^ pfu/g body weight). (B) ELISA of IFN‐α, IFN‐β, IL‐6, and TNF‐α in serum from *Zdhhc20* cKO and littermate mice 18 h after intraperitoneal injection of PBS or VSV. (C) q‐PCR analysis of *Ifnb1* and VSV RNA in the organs (spleen, lung, and liver) of the mice described in (B). (D) HE staining of lung sections from the mice described in (B). Scale bars, 50 µm. (E) Survival data for 8‐week‐old Zdhhc20 cKO mice and littermate controls (n=10) after intranasal injection of IAV (100 pfu per mouse). (F) ELISA of IFN‐β in serum from *Zdhhc20* cKO mice and littermate controls 3 days after intranasal injection of PBS or IAV. (G) HE staining of lung sections from the mice described in (F). Scale bars, 50 µm. (H) Survival data for mice (n = 8) fed a high palmitic acid or control diet for 3 weeks and infected intraperitoneally with VSV (5 × 10^7^ pfu/g body weight). (I) Survival data for mice (n = 8) fed a high palmitic acid or control diet for 3 weeks and infected intranasally with IAV (500 pfu per mouse). (J) *Zdhhc20^+/+^* or *Zdhhc20^−/−^* mice (n = 4) were intraperitoneally injected with PA (50 mg/kg) for 8 h and then infected intraperitoneally with VSV, after which IFN‐β levels in serum were detected by ELISA. (K) *Zdhhc20* ^+/+^ or *Zdhhc20^−/−^* mice (n = 4) were intraperitoneally injected with PA for 8 h and then infected intranasally with IAV, after which IFN‐β levels in serum were detected by ELISA. Statistical analysis was performed by one‐way ANOVA (B, C, F, J, and K). ns *P* >0.05, ^**^ *p* < 0.01, ^***^ *p* < 0.001, ^****^ *p* < 0.0001.

With a focus on the function of ZDHHC20 in macrophages, we generated myeloid‐specific *Zdhhc20‐*deficient mice. We found that *Zdhhc20* conditional knockout (cKO) mice were more susceptible to viral infection and showed increased lethality even when a sublethal dose of VSV was given (Figure 3A). *Zdhhc20*‐cKO mice consistently produced lower serum levels of IFN‐β and IFN‐α, as well as less IL‐6 and TNF‐α (Figure 3B). Compared with their littermates, *Zdhhc20*‐cKO mice displayed significantly decreased *Ifnb1* expression and elevated VSV loads in several organs (Figure 3C). Hematoxylin‐eosin (HE) staining revealed a severe pathological state characterized by massive infiltration of inflammatory cells and increased lung tissue damage in the absence of *Zdhhc20* (Figure 3D). To further prove the physiological function of ZDHHC20, we challenged cKO and WT mice with the influenza virus strain A/Puerto Rico/8/1934 H1N1 (PR8) through intranasal infection. Our data revealed similar results upon IAV infection to those obtained in the VSV infection model (Figure 3E‐G). As PA is a major component of palm oil and animal fat, we fed mice a short‐term high‐fat (mostly palm oil) diet. The data revealed that a palm oil‐rich diet protected mice against both VSV infection and IAV infection (Figure 3H,I) and increased IFN‐β production in *Zdhhc20^+/+^* mice but not in *Zdhhc20^−/−^* mice (Figure 3J,K). Taken together, both in vitro and in vivo data demonstrated that ZDHHC20 is important for the antiviral innate response by promoting type I IFN production in a PA‐dependent manner.

### 2.4 ZDHHC20 Catalyzes the S‐Palmitoylation of CMPK2 in Response to RNA Virus Infection

**FIGURE 4:**
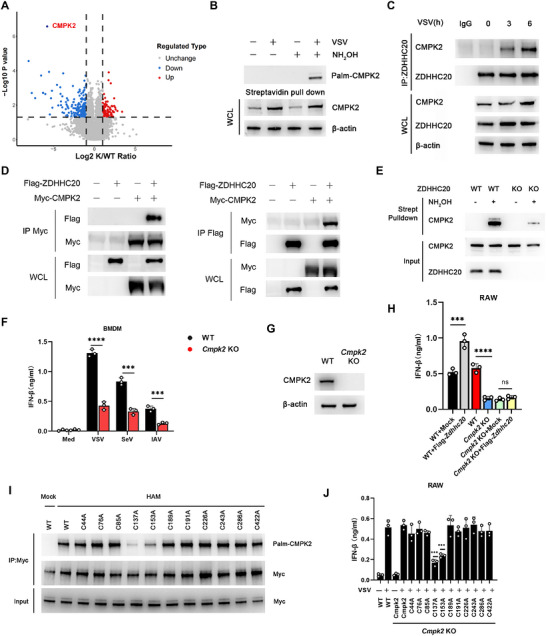
CMPK2 is S‐Palmitoylated by ZDHHC20. (A) Volcano plot showing changes in palmitoylated proteins. (B) The palmitoylation of endogenous CMPK2 derived from BMDMs infected with VSV for 6 h was analyzed by ABE assay and immunoblotting (IB) with or without hydroxylamine (NH_2_OH) treatment. (C) Co‐IP analysis of the interaction between ZDHHC20 and CMPK2 in untreated or VSV‐infected BMDMs. (D) Co‐IP analysis of the interaction between ZDHHC20 and CMPK2 in HEK293T cells co‐transfected with Flag‐tagged ZDHHC20 and Myc‐tagged CMPK2. (E) The palmitoylation of endogenous CMPK2 derived from WT and Zdhhc20 KO BMDMs infected with VSV for 6 h was analyzed by ABE assay. (F) ELISA of IFN‐β in the supernatants of *Cmpk2^+/+^* and *Cmpk2^−/−^* BMDMs untreated or infected with VSV, SeV, or IAV for 12 h. (G) Immunoblot analysis of CMPK2 in *Cmpk2^+/+^* and *Cmpk2^−/−^* RAW cells. (H) ELISA of IFN‐β in the supernatants of *Cmpk2^+/+^* and *Cmpk2^−/−^* RAW cells transfected with the indicated plasmids and then infected with VSV for 12 h. (I) The palmitoylation of CMPK2 derived from HEK293T cells transfected with the indicated plasmids was analyzed by ABE assay. (J) ELISA of IFN‐β in the supernatants of *Cmpk2^+/+^* and *Cmpk2^−/−^* RAW cells transfected with control or vectors encoding WT or mutant CMPK2, uninfected or infected with VSV for 12 h. Statistical analysis was performed by one‐way ANOVA (F, H, and J). ns *P* > 0.05, ^***^ *p* < 0.001, ^****^ *p* < 0.0001.

To identify the palmitoylated protein targets of ZDHHC20 that mediate enhanced antiviral innate immunity, we performed palmitoylation liquid chromatography‐mass spectrometry (LC‐MS/MS) analysis on macrophages after VSV infection. Compared with the WT group, the palmitoylation of CMPK2 in the *Zdhhc20^−/−^* group was significantly lower (Figure 4A). Furthermore, we observed that infection with VSV led to CMPK2 palmitoylation (Figure 4B) and used immunoprecipitation to show that both endogenous and exogenous ZDHHC20 interacted with CMPK2 and that this binding is stronger during VSV infection (Figure 4C,D). Notably, knocking out *Zdhhc20* decreased the palmitoylation of CMPK2 (Figure 4E), confirming that ZDHHC20 is the major endogenous CMPK2 palmitoyltransferase.

CMPK2 is a mitochondrial nucleotide monophosphate kinase that is needed to salvage dNTP synthesis and restricts the replication of multiple viruses, including flaviviruses [17] and coronaviruses [18]. Although *CMPK2* is an IFN‐stimulated gene (ISG), its role in macrophage IFN‐I production was previously unknown. We generated *Cmpk2* knockout mice and reported that IFN‐β secretion was impaired in *Cmpk2* ^−/−^ BMDMs compared with WT cells after infection with VSV, SeV, or IAV (Figure 4F). To determine whether ZDHHC20 promotes the type I IFN response via CMPK2 palmitoylation, we generated *Cmpk2*‐knockout RAW 264.7 cells (Figure 4G). We observed that overexpression of *Zdhhc20* did not further increase IFN‐I in *Cmpk2^−/−^* RAW cells (Figure 4H), indicating that ZDHHC20 is upstream of CMPK2 in antiviral immunity. Furthermore, ZDHHC20 promoted IFN‐I production and mouse survival during DNA virus HSV infection (Figure S3A–C), but CMPK2 had no such function (Figure S3D–F). Only RNA viruses triggered CMPK2 palmitoylation, and this modification was not observed upon DNA virus, bacterial, or fungal infection (Figure 4B; Figure S3G–J), indicating that the ZDHHC20‐CMPK2 axis selectively enhances the innate response against RNA virus infection and that ZDHHC20 has other substrates during DNA virus infection.

MS data identified 11 potential palmitoylation sites, and we constructed CMPK2 mutants by individually mutating these cysteine (C) residues to alanine (A) and found that compared with the WT, the C137 and C153 mutations weakened palmitoylation, whereas the other mutants largely retained this modification (Figure 4I). Thus, C137 and C153 are the predominant CMPK2 palmitoylation sites (Figure S4A). Next, we expressed either WT or mutant CMPK2 in RAW264.7 cells and found that mutant C137 and C153 notably decreased IFN‐β production in response to VSV infection (Figure 4J; Figure S4B). We also constructed cysteine (C) to serine (S) CMPK2 mutations and observed similar phenotypes (Figure S4C). Together, these results suggest that the ZDHHC20‐mediated increase in antiviral immunity is dependent on the palmitoylation of CMPK2.

### 2.5 The ZDHHC20‐CMPK2 Axis Promotes Antiviral Immunity via ddhCTP and MAVS

**FIGURE 5:**
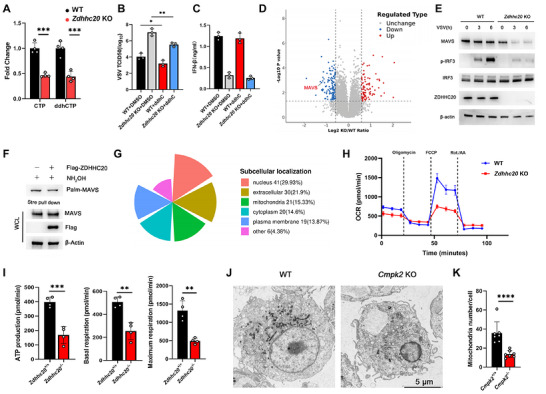
The ZDHHC20‐CMPK2 axis regulates ddhCTP and MAVS upon RNA virus infection. (A) LC‐MS/MS analysis of cellular metabolite levels in *Zdhhc20^+/+^* and *Zdhhc20^−/−^* BMDMs infected with VSV for 12 h. (B) Quantification of VSV replication in BMDMs. Cells were pretreated with ddhC for 24 h and then infected with VSV for 24 h, after which viral titers were determined by TCID50. (C) ELISA of IFN‐β in the supernatants of BMDMs pretreated with ddhC for 24 h and then infected with VSV for 12 h. (D) Volcano plot of proteins from *Zdhhc20^+/+^* and *Zdhhc20^−/−^* BMDMs infected with VSV for 6 h (n=3). (E) Immunoblot analysis of phosphorylated (p‐) or total proteins in the lysates of Zdhhc*20^+/+^* and *Zdhhc20^−/−^* BMDMs infected with VSV for the indicated time points. (F) The palmitoylation of MAVS derived from RAW264.7 cells transfected with the indicated plasmids was analyzed by ABE. (G) Pie chart showing the subcellular localization classification of the downregulated proteins in (D). (H) Oxygen consumption rate of *Zdhhc20^+/+^* and *Zdhhc20^−/−^* BMDMs infected with VSV for 6 h. (I) ATP production, basal respiration, and maximum respiration of cells in (H). (J) Representative electron microscopy images of *Cmpk2^+/+^* and *Cmpk2^−/−^* BMDMs infected with VSV for 6 h. Scale bar, 5 µm. (K) Quantitative analysis of mitochondria in BMDMs as described in (J). Statistical analysis was performed by unpaired two‐tailed Student's t tests (A, I, and K) and one‐way ANOVA (B). ^*^ *p* < 0.05, ^**^ *p* < 0.01, ^***^ *p* < 0.001, ^****^ *p* < 0.0001.

CMPK2 was previously reported to catalyze the ATP‐dependent phosphorylation of the monophosphates CMP, UMP, and dCMP to the corresponding diphosphates [19]. It was also shown to catalyze the formation of CDP and UDP to CTP and UTP, respectively. CMPK2 collaborates with viperin to provide sufficient CTP substrate for conversion to ddhCTP, [20] a metabolite that suppresses the replication of several coronaviruses through inhibition of viral RNA‐dependent RNA polymerase activity [18]. CMPK2 can also induce ribosome collision, leading to the activation of the integrated stress response and, consequently, translation inhibition [21]. In whole blood samples from COVID‐19 patients, ddhCTP intensity was strongly correlated with CMPK2 gene expression [22]. Since our data demonstrate that CMPK2 is palmitoylated during RNA virus infection, we hypothesized that ZDHHC20 regulates ddhCTP production. Metabolomics data confirmed this hypothesis (Figure 5A). To determine whether Zdhhc20 promotes antiviral immunity by promoting ddhCTP production, we treated WT and *Zdhhc20^−/−^* BMDMs with synthetic ddhC nucleoside, which crosses the plasma membrane and is converted to ddhCTP in the cytosol [20]. Treatment with ddhC repressed VSV replication (Figure 5B) but barely influenced IFN‐β levels (Figure 5C), suggesting that ZDHHC20 promotes IFN‐I production through a distinct mechanism.

To understand this mechanism, we determined changes in the proteome of *Zdhhc20* KO BMDMs using LC‐MS/MS. We noted that the expression of MAVS, a central adaptor in the antiviral immune pathway, was strongly depleted in the KO group compared with the WT group (Figure 5D). We validated these results using immunoblotting (Figure 5E). We also observed that palmitoylation‐deficient CMPK2 could not restore MAVS levels and IFN‐I signaling (Figure S4D). This effect on MAVS may explain why ZDHHC20 depletion affects IFN‐I production. However, ZDHHC20 barely influenced MAVS palmitoylation (Figure 5F).

Since deletion of *Cmpk2* in neurons leads to mitochondria deficiency, decreased mitochondrial proteins, and ATP production [23]. We wondered whether disruption of the ZDHHC20‐CMPK2 axis could cause mitochondrial dysfunction in macrophages during VSV infection. We found that 15% of proteins impacted in *Zdhhc20*‐KO samples localize to mitochondria (Figure 5G). We also used oxygen consumption rate (OCR) to evaluate the mitochondrial respiration capacity of BMDMs and found that *Zdhhc20* deletion decreased OCR (Figure 5H), including ATP production and respiratory capacity (Figure 5I). Moreover, *Cmpk2* deletion reduced the number of mitochondria (Figure 5J,K). In conclusion, the ZDHHC20‐CMPK2 axis protects mitochondria and maintains the abundance of MAVS during viral infection.

### 2.6 CMPK2 is Localized to Mitochondria and Interacts with HSPD1 to Maintain Mitochondrial Function

**FIGURE 6:**
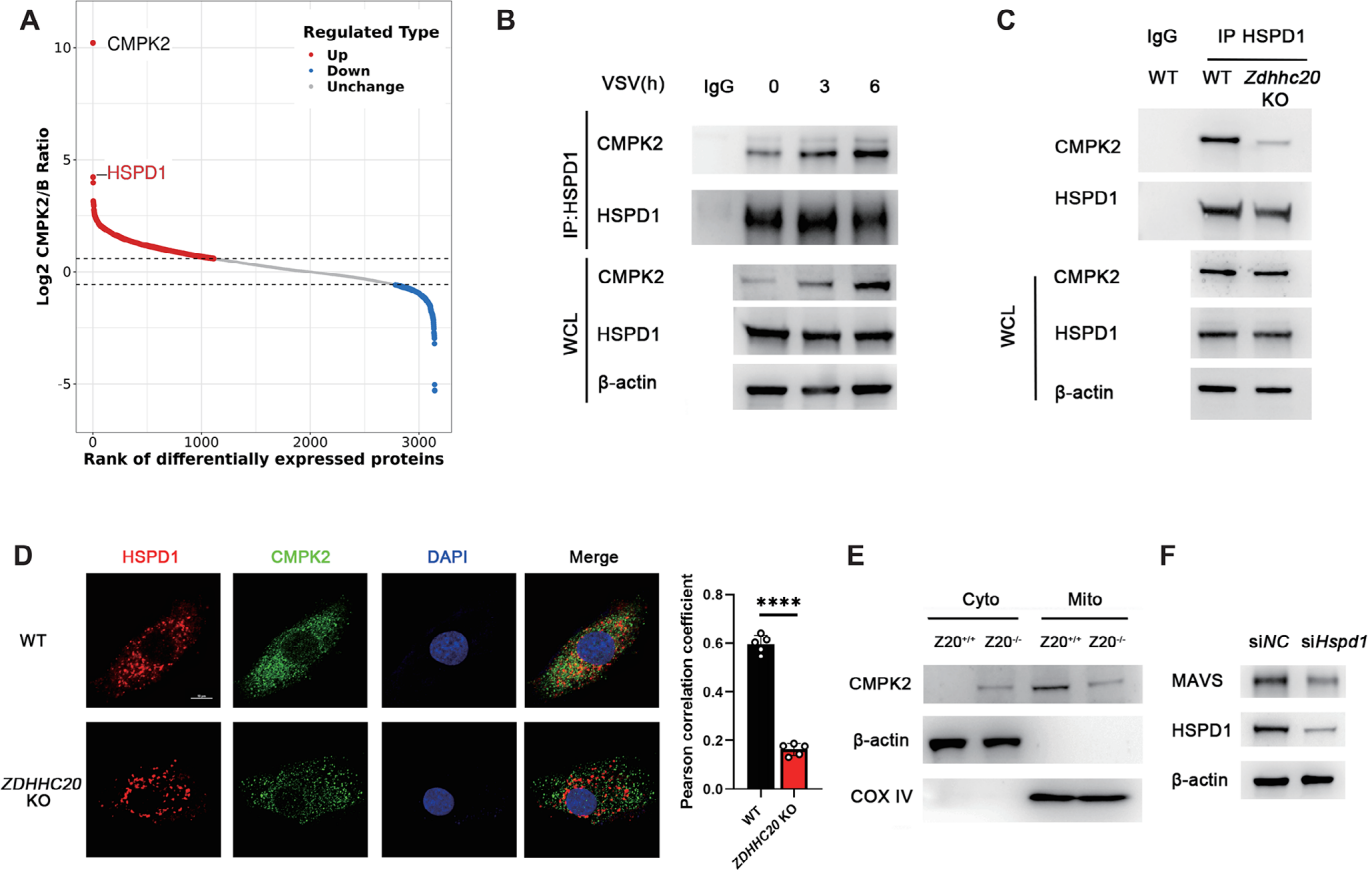
Palmitoylation promotes CMPK2 localization to mitochondria. (A) Proteins pulled down by anti‐Myc magnetic beads in A549 cells (transfected with Mock or Myc‐tagged CMPK2 plasmids for 48 h infected with VSV for 6 h were analyzed by IP‐MS. (B) Co‐IP analysis of the interaction between CMPK2 and HSPD1 in BMDMs infected with VSV for the indicated durations. (C) Co‐IP analysis of the interaction between CMPK2 and HSPD1 in *Zdhhc20^+/+^* and *Zdhhc20^−/−^* BMDMs infected with VSV for 6 h. (D) Immunofluorescence staining showing co‐localization of endogenous CMPK2 (green) and HSPD1 (red) in A549 cells infected with VSV for 6 h. DNA (blue) was stained with DAPI. Scale bar, 10 mm. Pearson's correlation coefficients of five double‐positive randomly selected cells were measured for fluorescence colocalization using ImageJ. (E) Immunoblot analysis of the subcellular localization of endogenous CMPK2 in BMDMs infected with VSV for 6 h. Protein levels were assessed in mitochondrial (Mito.) and cytosolic (Cyto.) fractions, which were verified using β‐actin and COX IV protein levels, respectively, as western blot loading controls. (F) Immunoblot analysis of total proteins in the lysates of BMDMs after transfection with control siRNA or Hspd1‐specific siRNA for 48 h and then infected with VSV for 6 h. Statistical analysis was performed by unpaired two‐tailed Student's t tests (D). ^*^ *p* < 0.05, ^**^ *p* < 0.01, ^***^ *p* < 0.001, ^****^ *p* < 0.0001.

To elucidate the mechanism through which the ZDHHC20‐CMPK2 axis affects the antiviral innate immune response and mitochondrial function, we performed an IP‐MS analysis to identify CMPK2‐binding partners upon VSV infection. As expected, CMPK2 was efficiently pulled down in the IP experiment, and HSPD1 (also known as HSP60) was the most abundant candidate protein among the proteins pulled down by CMPK2 (Figure 6A). Since HSPD1 perturbation results in mitochondrial dysfunction in immune cells [24], which is consistent with the phenotype of *Cmpk2* deficiency, we wondered whether CMPK2 maintained mitochondrial function through HSPD1. Endogenous Co‐IP demonstrated that CMPK2 physically interacted with HSPD1 in BMDMs and this interaction was enhanced upon VSV challenge (Figure 6B) and was maintained by ZDHHC20 (Figure 6C,D). Since HSPD1 is a well‐known mitochondrial marker, we further confirmed that ZDHHC20 maintained the mitochondrial localization of CMPK2 (Figure 6E). HSPD1 is a key mitochondrial chaperone protein that maintains mitochondrial homeostasis; therefore, we hypothesized that the binding of CMPK2 to HSPD1 is critical for maintaining mitochondrial function and the abundance of MAVS. As predicted, knockdown of *Hspd1* decreased MAVS (Figure 6F). Cycloheximide (CHX) chase assays showed that the degradation rates of MAVS were increased in both *Zdhhc20*‐KO and *Cmpk2*‐KO BMDM cells compared with that in WT cells (Figure S5), suggesting that CMPK2 palmitoylation stabilizes the MAVS protein, but the specific mechanism requires further investigation.

### 2.7 Thioesterase PPT1 Erases CMPK2 Palmitoylation and PPT1 Inhibitor Enhances Antiviral Immunity In Vivo

**FIGURE 7:**
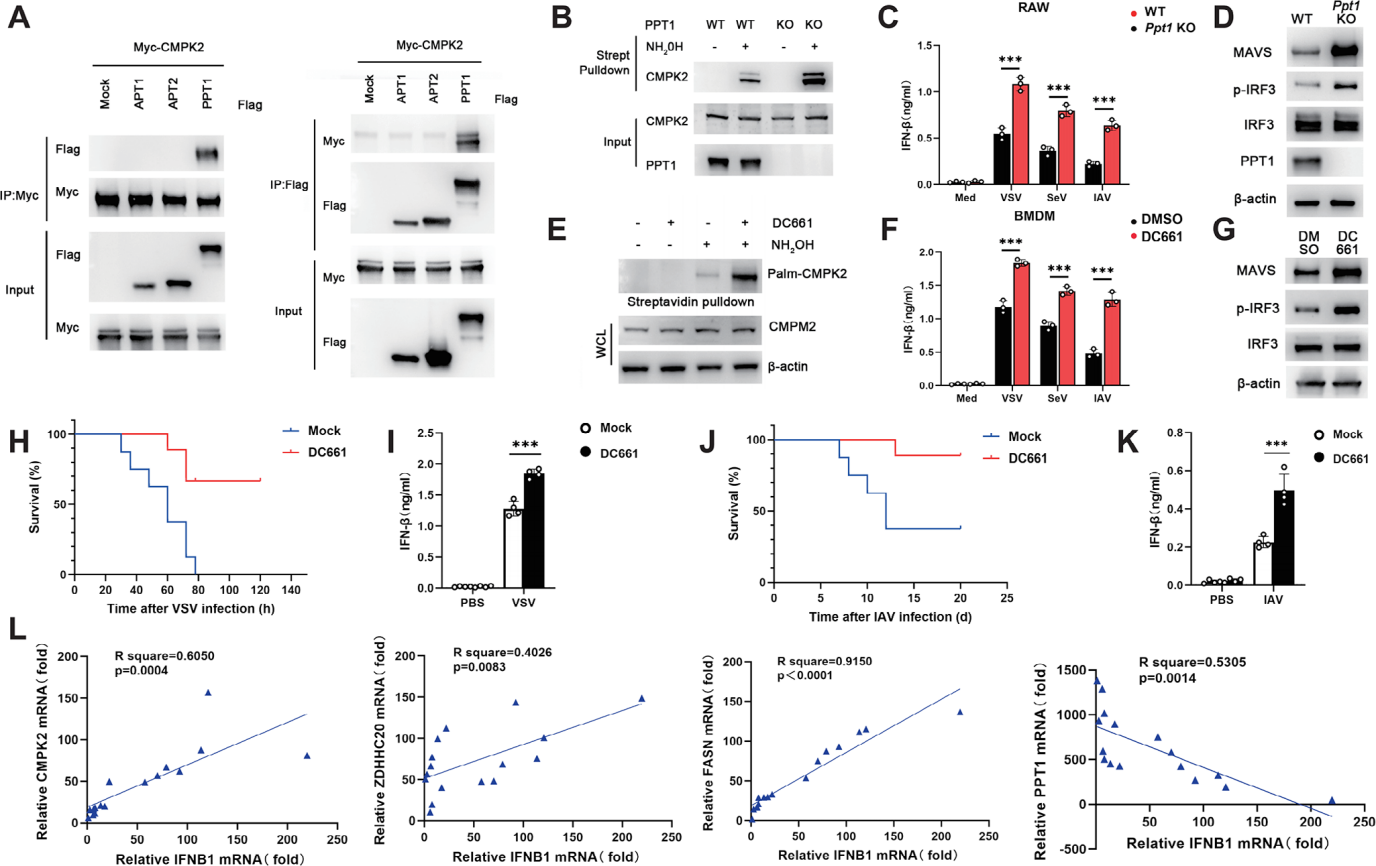
PPT1 depalmitoylates CMPK2, and a PPT1 inhibitor enhances the antiviral response. (A) Co‐IP analysis of the interaction between APTs and CMPK2 in HEK293T cells co‐transfected with Flag‐tagged APTs and Myc‐tagged CMPK2. (B) Palmitoylation of endogenous CMPK2 derived from WT and *Ppt1*‐deficient RAW264.7 cells infected with VSV for 6 h. (C) ELISA of IFN‐β in the supernatants of *Ppt1^+/+^* and *Ppt1^−/−^* RAW cells, untreated or infected with VSV, SeV, or IAV for 12 h. (D) Immunoblot analysis of phosphorylated (p‐) or total proteins in the lysates of *Ppt1^+/+^* and *Ppt1^−/−^* RAW cells infected with VSV for 6 h. (E) Palmitoylation of endogenous CMPK2 derived from BMDMs pretreated with DMSO or DC661 (5 µM) and then infected with VSV for 6 h was analyzed by ABE assay. (F) ELISA of IFN‐β in the supernatants from BMDMs pretreated with DMSO or DC661 and then untreated or infected with VSV, SeV, or IAV for 12 h. (G) Immunoblot analysis of phosphorylated (p‐) or total proteins in the lysates of BMDMs pretreated with DMSO or DC661 (5 µM) and then infected with VSV for 6 h. (H) Survival data for mice (n = 8) treated with DC661 (2 mg/kg) or control for 12 h and infected intraperitoneally with VSV (5 × 10^7^ pfu/g body weight). (I) ELISA of IFN‐β in serum from DC661 or control‐treated mice 18 h after intraperitoneal injection of PBS or VSV. (J) Survival data for mice (n = 8) treated with DC661 or control for 12 h and infected after intranasal injection of IAV (500 pfu per mouse). (K) ELISA of IFN‐β in serum from DC661 or control‐treated mice 3 days after intranasal injection of PBS or IAV. (L) Pearson correlations between CMPK2, ZDHHC20, FASN, and PPT1 mRNA levels and IFNB1 mRNA levels in COVID‐19 patient PBMCs. Statistical analysis was performed by one‐way ANOVA (C, F, I, and K). Spearman correlation analysis (L). ^***^ *p* < 0.001.

The opposing activities of palmitoyltransferases and their counteracting acyl‐protein thioesterases (APTs) govern the dynamic levels of reversible protein palmitoylation. To identify the depalmitoylase of CMPK2, we co‐expressed Flag‐tagged APT1, APT2, and PPT1 with Myc‐tagged CMPK2. Our data revealed that the thioesterase PPT1 specifically interacted with CMPK2 (Figure 7A). Since PPT1 has previously been studied for its link to cancer [25], its connection to viral infection remains unknown. We generated *Ppt1^−/−^* RAW cells using CRISPR/Cas9 technology and detected increased CMPK2 palmitoylation (Figure 7B) and IFN‐I production (Figure 7C) in *Ppt1*‐deficient RAW cells. Consistent with these findings, the expression of MAVS and the phosphorylation of interferon regulatory factor 3 (IRF3) were also increased upon VSV infection of *Ppt1^−/−^* cells (Figure 7D). Furthermore, we treated BMDMs with the PPT1‐specific molecular inhibitor DC661 [26]. As expected, DC661 increased CMPK2 palmitoylation (Figure 7E) and components of the IFN‐I pathway (Figure 7F,G). We also observed that the antiviral effects of DC661 treatment were significantly attenuated in *Cmpk2^−/−^* BMDMs (Figure S6), indicating that DC661 enhances the antiviral response through targeting CMPK2.

Next, we evaluated whether this PPT1 inhibitor can enhance innate immunity against viral infection in vivo. Notably, DC661 treatment increased IFN‐I induction and the survival of mice infected with high doses of VSV and IAV (Figure 7H–K). These findings suggest that PPT1 erases CMPK2 palmitoylation. Thus, targeting PPT1 could provide a promising therapeutic strategy to boost innate immunity against RNA virus infection. Finally, to determine the clinical significance of our findings, we assessed the expression of CMPK2, ZDHHC20, FASN, PPT1, and IFNB1 in peripheral blood mononuclear cells (PBMCs) from COVID‐19 patients. We found that high expression of IFNB1 in PBMCs was closely related to high CMPK2, ZDHHC20, and FASN expression and low PPT1 expression (Figure 7L).

### 2.8 Palmitic Acid‐Mediated CMPK2 Palmitoylation Promotes Antiviral Innate Immunity

## 3 Conclusions and Discussion

**FIGURE 8:**
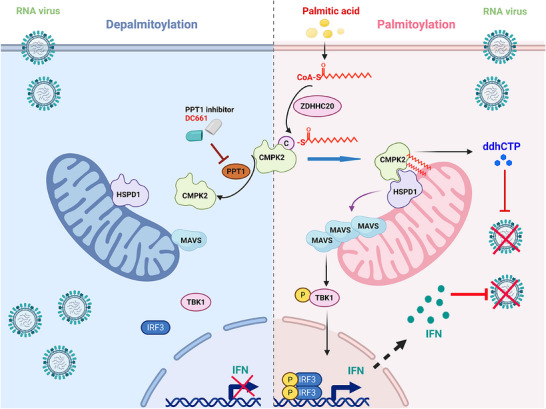
Working model. Graphical abstract created with BioRender depicting the mechanism by which PA promotes host antiviral immune response.

The immune system and metabolic system are highly integrated, and metabolites are emerging as fundamentally important signaling molecules in the immune response. Here, we revealed a previously unknown mechanism through which PAs drive antiviral immunity against RNA viruses. We identified both the target of palmitoylation, CMPK2, as well as the writer, ZDHHC20, and the eraser, PPT1, of this modification (Figure 8). The interplay between these factors underpins the response of macrophages to RNA virus infection and hinges on a mitochondrial mechanism that also affects the robust MAVS response.

The function of macrophages is closely regulated by cellular metabolic levels, [27] including the levels of metabolites of glycolysis, mitochondrial respiration, lipid synthesis, and lipolysis [28]. By screening a metabolism‐modulating chemical library, we identified PA as a previously unknown driver of antiviral immunity against RNA viruses. The covalent binding of lipids to proteins, which is termed protein lipidation, plays an essential role in the regulation of protein trafficking, localization, stability, conformation, interactions, and signal transduction. Our data reveal that the PA‐mediated reversible S‐palmitoylation of CMPK2 is a key activator of innate immunity against RNA virus infection.

Surprisingly, to our knowledge, previous studies have not reported the modification of CMPK2, a rate‐limiting enzyme in CTP synthesis. Recent advances have demonstrated the multilayered effects of palmitoylation on various cellular processes, including cell death [29], inflammation [30], autophagy [31], and metabolic disease [32]. Emerging evidence has revealed the inextricable link between S‐palmitoylation and infectious diseases [33]. Taking SARS‐CoV‐2 as an example, the palmitoylation of both the viral spike protein and the receptor ACE2 is vital for viral fusion and infection [34]. However, how palmitoylation affects host proteins in macrophages to clear viruses has been elusive. In this study, palmitoylation maintained CMPK2 mitochondrial localization and mitochondrial homeostasis in macrophages during RNA virus infection.

Indeed, lipid reprogramming affects other aspects of immune cell function during viral infection [35], providing energy to macrophages, facilitating viral clearance by affecting membrane dynamics, signal transduction, and interferon secretion [36]. In addition, IFN‐I stimulation increases the expression of cholesterol‐25‐hydroxylase (CH25H), which can convert cholesterol to 25‐hydroxycholesterol (25‐HC) [37]. 25‐HC is augmented in macrophages after viral infection and works as a natural antiviral agent to combat a broad range of viruses, including Zika virus [38] and SARS‐CoV‐2 [39]. Serum levels of palmitic acid may negatively correlate with disease severity after H7N9 infection [40]. Our data revealed a positive correlation between the expression of CMPK2, ZDHHC20, and IFNB1 in COVID‐19 patient PBMCs, indicating that the palmitoylation of CMPK2 augmented antiviral immunity. Our findings greatly expand our understanding of lipid metabolism and IFN‐I signaling, which form a feedback loop to control viral infection cooperatively.

We specifically revealed that CMPK2 C137 and 153 were subject to S‐palmitoylation by ZDHHC20 in RNA virus‐infected cells and anchored CMPK2 to mitochondria. Mitochondrial localization controlled by ZDHHC20 is vital for enzyme catalysis to produce the downstream metabolite ddhCTP, which directly inhibits RNA viral replication. Moreover, ZDHHC20 maintained the abundance of MAVS to produce IFN‐I. As a central receptor of antiviral immunity, MAVS undergoes various PTMs, including ubiquitination [41], methylation [42], and palmitoylation [43]. In our study, instead of direct modification, ZDHHC20 functioned as a scaffold, assisting CMPK2 binding to HSPD1 and thus maintaining MAVS. However, the underlying mechanisms through which CMPK2 affects the function of HSPD1 in mitochondrial homeostasis remain to be further investigated. In conclusion, we reported that reversible palmitoylation of CMPK2 activated antiviral immunity against RNA viruses through IFN‐dependent and IFN‐independent pathways.

Our data revealed that ZDHHC20 promoted innate immunity against both RNA and DNA viruses, but that CMPK2 barely influenced IFN‐I upon HSV infection. In addition, the palmitoylation of CMPK2 was induced only by RNA viruses, suggesting that ZDHHC20 might catalyze the modification of other substrates upon DNA virus infection. Previous studies reported that only ddHCTP repressed RNA virus replication and that MAVS is downstream of the RNA sensor RIG‐I, not cGAS‐STING. Taken together, these findings might explain why the ZDHHC20‐CMPK2 axis selectively regulates antiviral immunity against RNA viruses.

Targeting lipid metabolism in macrophages has been proven to be effective in animal infection models. For example, inhibition of the triglyceride‐synthesizing enzyme DGAT1 blocks hepatitis C virus particle formation [44]. Owing to the vital role of CMPK2 palmitoylation in antiviral immunity, developing activators of ZDHHC20 could offer a potential strategy to cure infectious diseases. Considering the technical challenges to achieve this goal, an alternative is to block depalmitoylases. For instance, ML349 (an inhibitor of APT2) blocks the depalmitoylation of STAT3 and alleviates DSS‐induced colitis [30]. In this work, we revealed that DC661, which inhibits PPT1, promoted IFN‐I production by maintaining CMPK2 palmitoylation during RNA virus infection. DC661 treatment prolonged the survival of mice after infection and dampened the viral burden. Thus, PA and DC661 have potential for future translational studies aimed at the clinical treatment of viral infection.

The interplay between macrophage lipid metabolism and the antiviral response constitutes a highly dynamic regulatory network, consisting of changes in enzyme activity, membrane structure remodeling, and signaling molecule conversion. A deeper understanding of this network not only helps explain the tissue specificity of viral infections but also lays the foundation for the development of “immune‐metabolic” dual‐target drugs.

## 4 Experimental Section

### 4.1 Animal Studies

C57BL/6 mice (6–8 weeks of age) were obtained from Shanghai Joint Ventures Sipper BK Experimental Animal Company. *Zdhhc20*‐KO (S‐KO‐14774), *Zdhhc20*‐flox (S‐CKO‐16372), and *Cmpk2*‐KO (S‐KO‐16099) mice were obtained from Cyagen Biosciences (China). *Lyz2*‐Cre mice (JAX 004781) were obtained from Jackson Laboratory. All the animals were bred and housed under SPF facilities at Naval Medical University (Shanghai, China). The experiments were performed in accordance with the NIH Guide for the Care and Use of Laboratory Animals. All animal protocols were approved by the Animal Care and Use Committees of the Institute of Laboratory Animal Science of the Chinese Academy of Medical Sciences (ACUC‐A01‐2023‐001).

### 4.2 Human Sample Collection

Peripheral blood samples were collected from 16 SARS‐CoV‐2 Omicron BA.1‐infected individuals (confirmed by RT‐PCR test or second‐generation sequencing), and the study was approved by the Ethics Committee of Nankai University (No. 2022N045KY). Written informed consent forms were signed before sample collection from all individuals.

### 4.3 Cells Lines

The HEK293T, RAW 264.7, and THP‐1 cell lines were obtained from the American Type Culture Collection (ATCC). PMs were isolated from mouse peritoneal cavities 4 days after intraperitoneal injection of 3% fluid thioglycollate medium (BD Bioscience). Cells were cultured in DMEM supplemented with 10% (v/v) fetal bovine serum (FBS) (GIBCO). BMDMs were differentiated from bone marrow cells and cultured in RPMI 1640 medium supplemented with 10% (v/v) FBS in the presence of recombinant 20 ng/ml murine M‐CSF (PeproTech).

### 4.4 Antibodies

ZDHHC20 antibody (Cat# sc‐518217) and MAVS antibody (Cat# sc‐166583) were purchased from Santa Cruz Biotechnology. Normal rabbit IgG (Cat# 2729), Myc‐Tag antibody (Cat# 2276), DYKDDDDK‐Tag antibody (Cat# 14793), IRF‐3 antibody (Cat# 4302), and phospho‐IRF‐3 (Ser396) antibody (Cat# 4947) were purchased from Cell Signaling Technology. CMPK2 antibody (Cat# PA5‐34461) was purchased from Thermo Scientific. Antibodies against HSP60 (Cat# 66041‐1‐Ig), PPT1 (Cat# 29653‐1‐AP), and beta‐actin (Cat# 66009‐1‐Ig) were purchased from Proteintech. CMPK2 antibody (Cat# PSI‐7063) was purchased from ProSci. AbSmart Goat Anti‐Mouse lgG HRP (Cat# M21004) and AbSmart Mouse Anti‐Rabbit lgG HRP (Cat# M21006) were purchased from Abmart.

### 4.5 Virus

Vesicular stomatitis virus (VSV, Indiana strain) was amplified by infection of a monolayer of HEK293T cells. Sendai virus (SeV, a gift from Dr. Bin Sun, Shanghai Institutes for Biological Sciences, Chinese Academy of Sciences, China) and influenza A/PR/8/1934 virus (ATCC) were propagated in SPF chicken eggs. Herpes simplex virus type 1 (HSV‐1, Kos strain) was a gift from Dr. Qihan Li (Chinese Academy of Medical Sciences, China).

### 4.6 High‐Throughput Metabolism‐Modulating Chemical Library Screening

Peritoneal macrophages were seeded onto 48‐well cell culture plates overnight. A chemical library (TargetMol) of 1471 metabolic disease‐related compounds (including metabolites and inhibitors/agonists of metabolic enzymes) and DMSO controls were added to the wells. Compounds (10 mM in DMSO) were diluted to a final concentration of 20 µM on the screen. At 48 h posttreatment, the cells were infected with VSV for 24 h. IFN‐β in the supernatants was then detected by ELISA, and the results were normalized to DMSO‐treated wells.

### 4.7 RT‐PCR and qRT‐PCR Analysis

Total RNA was extracted from cultured cells and tissues using either TRIzol reagent (Invitrogen) or miRNeasy Micro Kit (QIAGEN). Equal amounts of RNA were reverse‐transcribed into cDNA using a ReverTra Ace Transcription Kit (Toyobo). Diluted cDNAs were subjected to q‐PCR analysis using SYBR Green Premix Pro Taq HS qPCR Kit (Accurate Biology), followed by melting curve analysis. The data were normalized to the level of β‐actin expression in each sample. The 2‐ΔΔC(t) method was used to calculate relative expression changes. The qPCR primers used for detecting the expression of the indicated RNAs are listed in Table S1.

### 4.8 Gene Knockout by CRISPR/Cas9

We generated *Cmpk2 ^−/−^, Ppt1^−/−^* RAW264.7 cells and *ZDHHC20^−/−^* A549 cells using the CRISPR‐Cas9 system. In brief, guide RNA sequences were selected through the http://crispr.mit.edu website and cloned into CRISPR‐Cas9 RNP (provided by AisenGene Bioscience). RNP complexes were electrotransfected into cells using the Neon transfection system (Thermo Scientific). After two days, single colonies were transferred to 96‐well plates. To determine the presence of insertions or deletions in targeted clones, genomic DNA was isolated using a Quick‐DNA Miniprep kit (Zymo Research) and PCR amplification. Genomic DNA was retrieved from the single clones and sequenced by Sanger sequencing. The sequences of the sgRNAs are listed in Table S2.

### 4.9 Flow Cytometric Analysis

Single‐cell suspensions from mouse spleens were stained with fluorescent‐conjugated primary antibodies. Cells were detected using a SONY ID7000, and the data were analyzed using FlowJo software (Tree Star).

### 4.10 Immunoblot Assay and Co‐Immunoprecipitation Assay

Cells were harvested and lysed using cell lysis buffer (Cell Signaling Technology) supplemented with a protease inhibitor cocktail set (Millipore Sigma). Protein concentrations were determined with the BCA Protein Assay reagent (Thermo Scientific), and adjusted lysates were resolved by SDS‐PAGE. The separated proteins were transferred to a nitrocellulose membrane and detected with appropriate antibodies. HEK293T cells were co‐transfected with plasmids for 36 h, followed by harvesting and lysis with cell lysis buffer. The cell lysates were incubated with anti‐Flag (Sigma Aldrich) or anti‐Myc magnetic beads (Thermo Scientific) at 4°C for 4 h. The beads were subsequently washed two times with NETN900 (20 mM Tris‐HCl, pH 8.0; 0.1 mM EDTA; 0.5% NP‐40; 900 mM NaCl) and three times with NETN100. Washed beads were then boiled with loading buffer for 10 min and subjected to immunoblotting. For endogenous IP, PMs were lysed with cell lysis buffer. Equalized extracts were incubated with the antibody for 8 h at 4°C and with protein A/G agarose beads (Santa Cruz) for 2 h. Agarose beads were subsequently washed two times with NETN600 and three times with NETN100. Washed beads were then boiled with loading buffer for 10 min and subjected to immunoblotting.

### 4.11 Mass Spectrometry (MS) Analysis of S‐Palmitoylation

Samples were taken from −80°C, weighed into a mortar, ground to powder with liquid nitrogen, and mixed with 4 × volume lysis buffer (1% SDS, 1% protease inhibitor, 25 mM NEM). After being sonicated in an ice‐water bath in the dark for 1 h, the lysate was centrifuged at 12 000 × g for 10 min at 4°C. The supernatant was mixed with 4 × volume pre‐cooled acetone, precipitated at −20°C for 4 h, and centrifuged again, after which the protein pellet was washed three times with pre‐cooled acetone and then resuspended in lysis buffer. For trypsin digestion, equal amounts of protein were adjusted to the same volume, treated with 0.7 M hydroxylamine and 1 mM HPDP‐biotin, precipitated with acetone, washed, and 100 mM ammonium bicarbonate was added, and the mixture was sonicated and digested with trypsin (1:50 w/w) overnight at 37°C. For enrichment, 0.5 mg of peptide was bound to prewashed resin in IP buffer, washed with IP wash buffer, eluted with TCEP‐containing buffer, treated with 30 mM IAM, vacuum‐dried, desalted via C18 ZipTips, and finally subjected to LC‐MS/MS. Peptides were ionized at 1.6 kV and analyzed by timsTOF HT in dia‐PASEF mode. MS was conducted by PTM Bio (China).

### 4.12 4D‐FastDIA Proteomics

After retrieving samples from −80°C, 4 volumes of lysis buffer containing 1% SDS and 1% protease inhibitor were added. After lysis and centrifugation, the supernatant was transferred to a new tube for BCA protein quantification. Equal protein amounts were adjusted to uniform volumes with lysis buffer, precipitated by sequential addition of 1 and 4 volumes of pre‐chilled acetone, incubated at −20°C for 2 h, centrifuged at 4500 × g for 5 min, washed 3 times with cold acetone, dried, resuspended in 100 mM ammonium bicarbonate via sonication, digested overnight with trypsin (1:50 w/w), reduced with 5 mM DTT at 37°C for 60 min, and alkylated with 11 mM IAA in the dark for 45 min. The resulting peptides were dissolved in mobile phase A (0.1% formic acid, 2% acetonitrile), separated using an Easy‐nLC1000 UHPLC system with a gradient of 6%–24% B (0.1% formic acid in acetonitrile) for 14 min, 24%–35% B (14–16 min), 35%–90% B (16–18 min), and 90% B (18–20 min) at 500 nL/min, ionized via a capillary source, and analyzed by timsTOF Pro MS using dia‐PASEF mode with a 300–1500 m/z MS1 scan range, 20 PASEF MS2 scans, and 7 m/z isolation windows from 400–850 m/z. Data are processed with DIA‐NN v1.8 against Mus musculus 10090 SP 20231220. fasta concatenated with a reversed decoy database, specifying trypsin/P cleavage with up to 1 missed cleavage, fixed modifications of N‐terminal Met excision and Cys carbamidomethylation, and an FDR threshold of <1%.

### 4.13 Acyl‐Biotin Exchange (ABE) Assay

Endogenous CMPK2 palmitoylation analysis was performed as previously reported [45]. Briefly, the cell pellets were resuspended in blocking buffer containing 100 mM N‐ethylmaleimide (NEM) and 1 mM PMSF. The supernatants were then rotated at 4°C for 3 h and precipitated with methanol for 2 h at 80°C. The pellets were dissolved in hydroxylamine (HAM)‐containing binding buffer (100 mM HEPES, pH 7.5, 1.0 mM EDTA, and 1% SDS) and then treated with biotin‐BMCC (5 mM) at room temperature for 1 h. Subsequently, the labeled proteins were enriched using streptavidin magnetic beads (Thermo Scientific) at room temperature with rotation for 3 h. The beads were subsequently washed 3 times with wash buffer (Tris‐buffered saline containing 0.1% Tween‐20 detergent), and the proteins were eluted with elution buffer (10 mM EDTA, pH 8.2 and 95% formamide) at 95°C for 15 min. Finally, the proteins were separated by SDS‐PAGE for western blotting.

To examine the palmitoylation of exogenous proteins, 293T cells were transfected with Myc‐tagged CMPK2‐WT or mutants for 48 h and then harvested in lysis buffer, followed by incubation with anti‐c‐Myc magnetic beads (Pierce) overnight at 4°C. The palmitoylation of CMPK2 was detected using an IP‐ABE Palmitoylation Kit (Aimsmass) according to the manufacturer's instructions.

### 4.14 Statistical Analysis

The statistical significance of comparisons between two groups was analyzed with a two‐tailed Student's t‐test. For comparisons among more than two groups, one‐way ANOVA with multiple comparisons was used. Differences were considered to be statistically significant when *p*‐values were less than 0.05. ^*^ *p* < 0.05, ^**^ *p* < 0.01, ^***^ *p* < 0.001, ^****^ *p* < 0.0001.

## Author Contributions

X.C. and Y.W. designed the experimental approach and supervised the study; Y.W., Z. C., and Y.Z. performed the experiments; Z.L. provided COVID‐19 patient samples; Y.W. and X.C. analyzed the data and wrote the paper.

## Conflicts of Interest

The authors declare no conflicts of interest

## Supporting information




**Supporting File**: advs75209‐sup‐0001‐SuppMat.docx.

## Data Availability

The data that support the findings of this study are available from the corresponding author upon reasonable request.
