# Supplementary material for: Palmitic Acid Promotes Antiviral Innate Immunity via ZDHHC20‐Mediated CMPK2 Palmitoylation
Source: Adv Sci (Weinh). 2026 Apr 21:e75209. Online ahead of print. doi: 10.1002/advs.75209 (PMC13334629; doi:10.1002/advs.75209)

Supporting Information

**Palmitic acid promotes antiviral innate immunity via ZDHHC20-mediated CMPK2 palmitoylation**

Yujia Wang*, Zenghui Cui, Yunkai Zhang, Zhiqing Li and Xuetao Cao*

**Supplementary Figure 1**

#
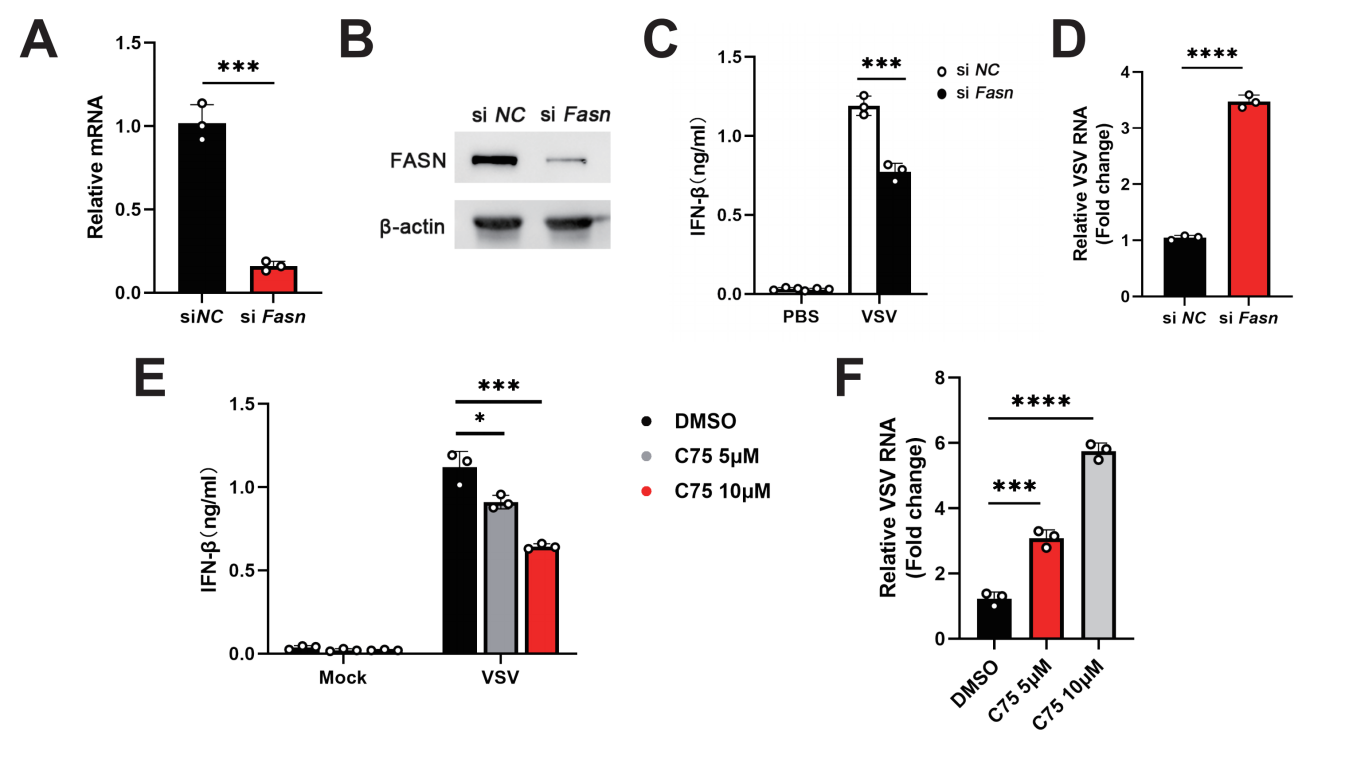


**Figure S1 FASN facilitates antiviral immune response.**

1. q-PCR analysis of *Fasn* expression in BMDMs 48 h after transfection with control or *Fasn*-specific siRNA.
2. Immunoblot analysis of *FASN* expression in BMDMs treated as described in (A).
3. ELISA of IFN-β in the supernatants of BMDMs treated as described in (A) and then infected with VSV for 12 h.
4. q-PCR analysis of VSV RNA in BMDMs as described in (C).
5. ELISA of IFN-β in the supernatants of BMDMs pretreated with DMSO or C75 and then infected with VSV for 12 h.
6. q-PCR analysis of VSV RNA in BMDMs as described in (E).

Statistical analysis was performed by unpaired two-tailed Student's t-test (A, D); one-way ANOVA (C, E and F). **P* < 0.05, ****P* < 0.001, *****P* < 0.0001.

**Supplementary Figure 2**

**
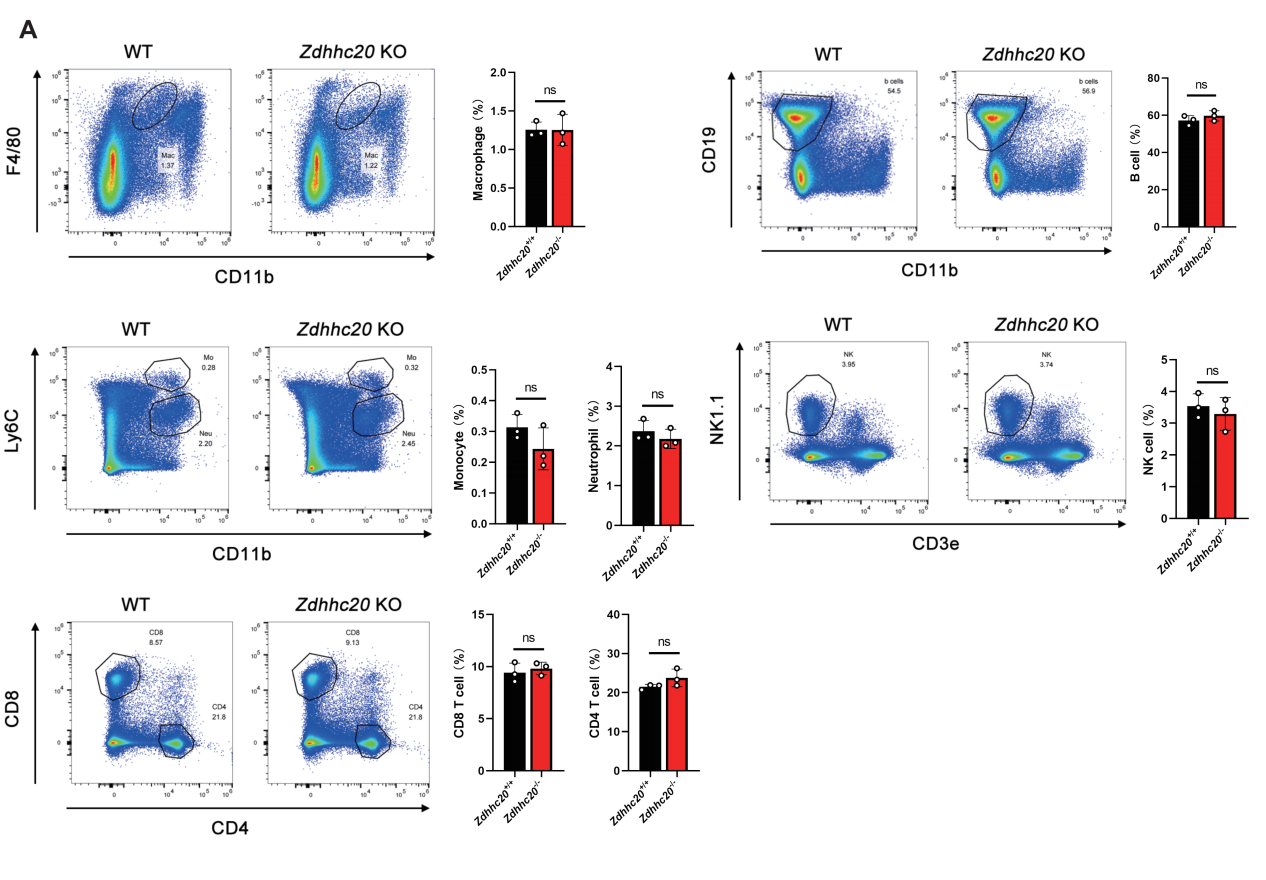
**

**Figure S2 ZDHHC20 has no influence on the development of immune cells.**

FACS analysis of F4/80, CD11b, Ly6c, CD3[ε](https://baike.baidu.com/item/%CE%B5/2842514), CD8, CD4, CD19, and NK1.1 expression in lymphocytes from spleens of *Zdhhc20*^+/+^ and *Zdhhc20*^-/-^ mice.

Statistical analysis was performed by unpaired two-tailed Student's t-test. ns, not significant.

**Supplementary Figure 3**


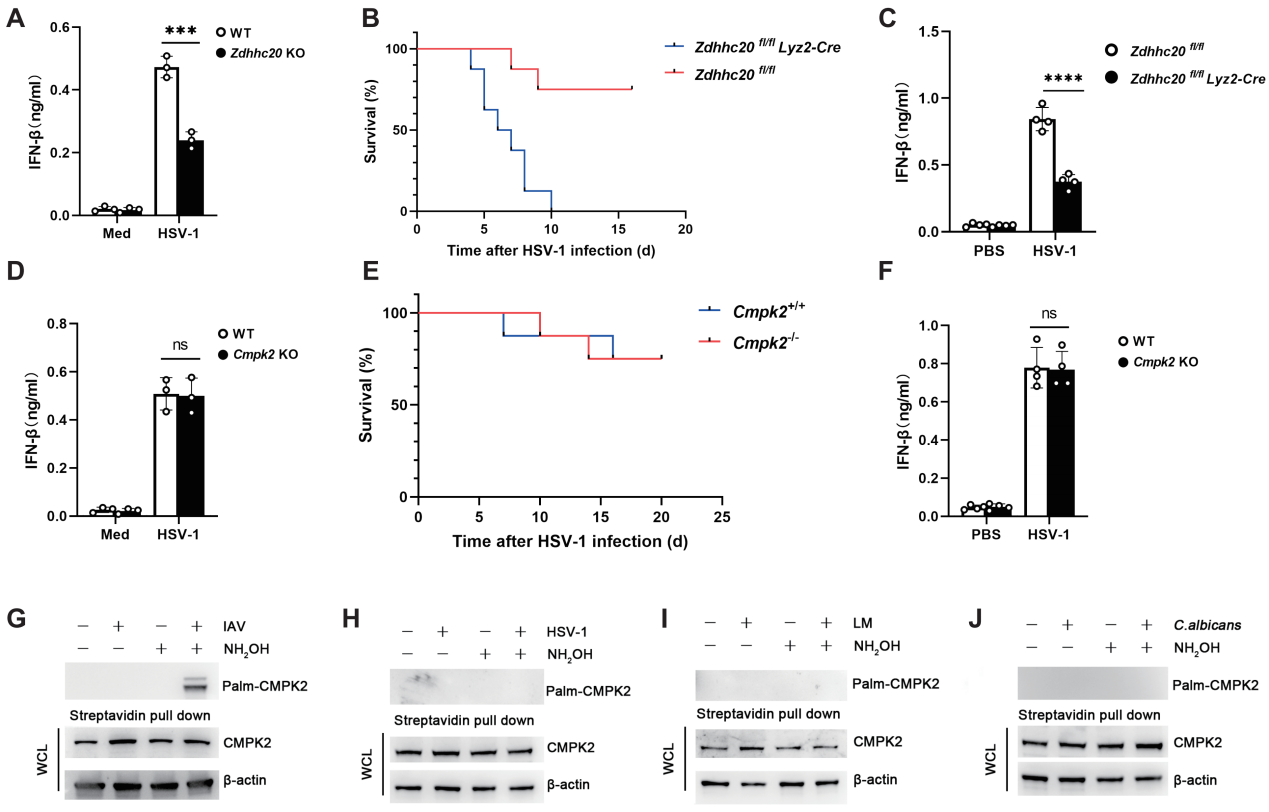


**Figure S3 ZDHHC20 selectively catalyzes CMPK2 palmitoylation during RNA virus infection.**

1. ELISA of IFN-β in supernatants of *Zdhhc20*^+/+^ and *Zdhhc20*^-/-^ BMDMs untreated or infected with HSV-1 for 9 h.
2. Survival data for 8-week-old *Zdhhc20* cKO and littermate mice (n=8 per group) after intraperitoneal injection of HSV-1.
3. ELISA of IFN-β in serum from *Zdhhc20* cKO and littermate mice 6 h after intraperitoneal injection of PBS or HSV-1.
4. ELISA of IFN-β in supernatants of *Cmpk2*^+/+^ and *Cmpk2*^-/-^ BMDMs untreated or infected with HSV-1 for 9 h.
5. Survival data for 8-week-old *Cmpk2* KO and littermate mice (n=8 per group) after intraperitoneal injection of HSV-1.
6. ELISA of IFN-β in serum from *Cmpk2* KO and littermate mice 6 h after intraperitoneal injection of PBS or HSV-1.
7. Palmitoylation of endogenous CMPK2 derived from BMDMs infected with IAV was analyzed by ABE assay, with or without NH_2_OH treatment.
8. Palmitoylation of endogenous CMPK2 derived from BMDMs infected with HSV-1.
9. Palmitoylation of endogenous CMPK2 derived from BMDMs infected with *Listeria monocytogenes*.
10. Palmitoylation of endogenous CMPK2 derived from BMDMs infected with *Candida Albicans*.

Statistical analysis was performed by one-way ANOVA(A, C, D and F). ns *P*>0.05, ****P* < 0.001, *****P* < 0.0001.

**Supplementary Figure 4**


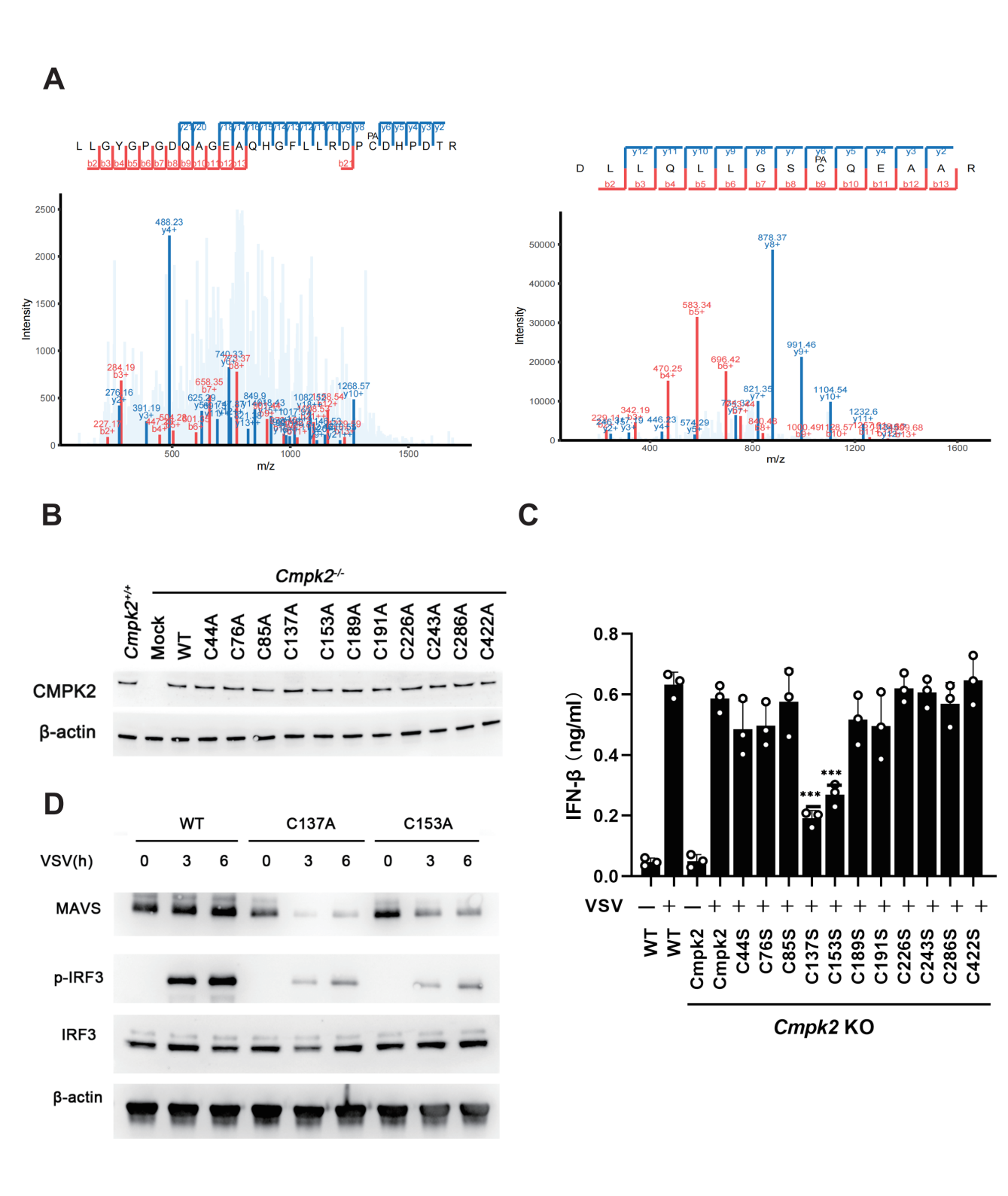


**Figure S4 Identification of modified cysteine residues in CMPK2.**

1. Mass spectrometry analysis of palmitoylation modification at C137 and C153 sites of CMPK2.
2. Immunoblot analysis of proteins in lysates of *Cmpk2*^+/+^ and *Cmpk2*^-/-^ RAW cells transfected with control or vectors encoding WT or mutant CMPK2.
3. ELISA of IFN-β in the supernatants of *Cmpk2*^+/+^ and *Cmpk2*^-/-^ RAW cells transfected with control or vectors encoding WT or mutant CMPK2, uninfected or infected with VSV for 12 h.
4. Immunoblot analysis of phosphorylated (p-) or total proteins in the lysates of indicated RAW cells infected with VSV for the indicated time points.

Statistical analysis was performed by one-way ANOVA (C). ****P* < 0.001.

**Supplementary Figure 5**


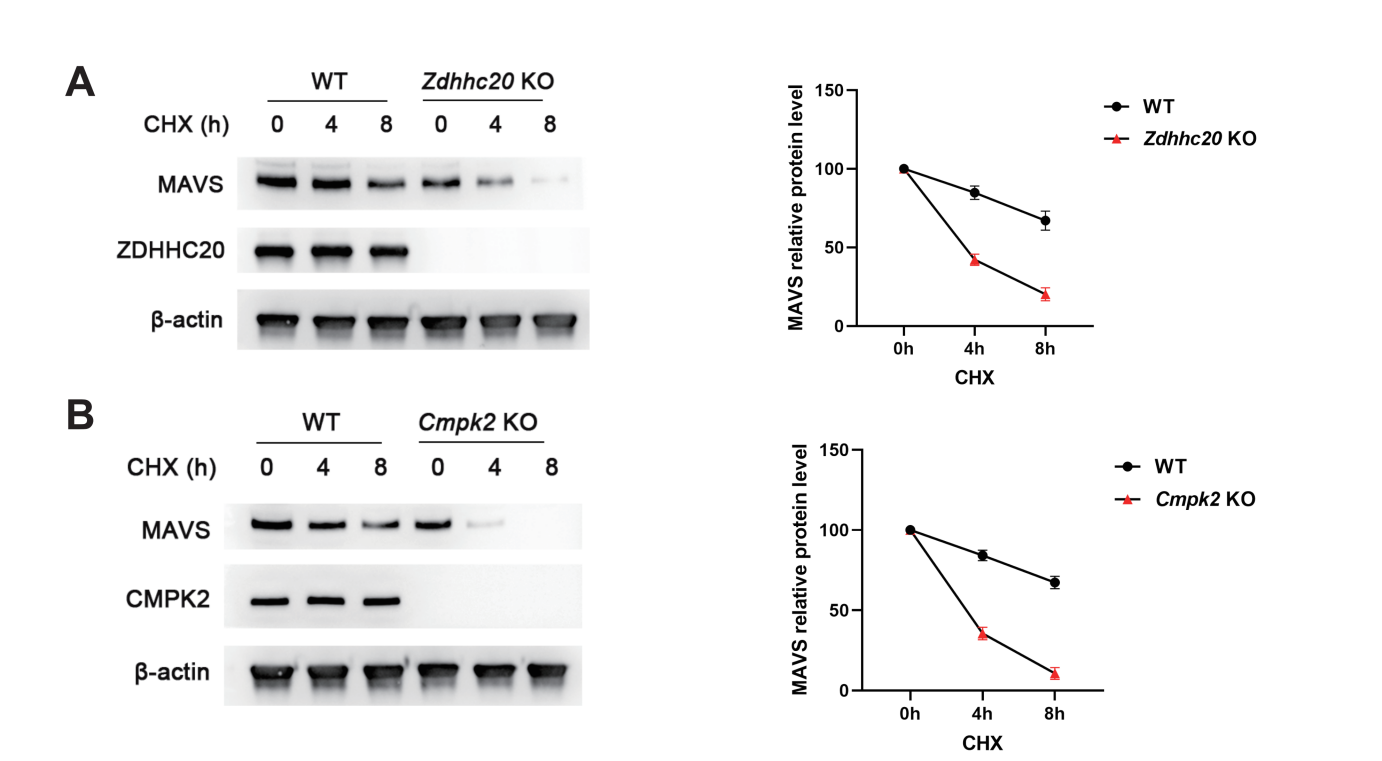


**Figure S5 CMPK2 palmitoylation stabilizes the MAVS protein.**

1. Immunoblot analysis of proteins in lysates of VSV infected *Zdhhc20*^+/+^ and *Zdhhc20*^-/-^ BMDMs treated with CHX for the indicated time points. Relative expression level of MAVS is calculated by the ImageJ.
2. Immunoblot analysis of proteins in lysates of VSV infected *Cmpk2*^+/+^ and *Cmpk2*^-/-^ BMDMs treated with CHX for the indicated time points. Relative expression level of MAVS is calculated by the ImageJ.

**Supplementary Figure 6**


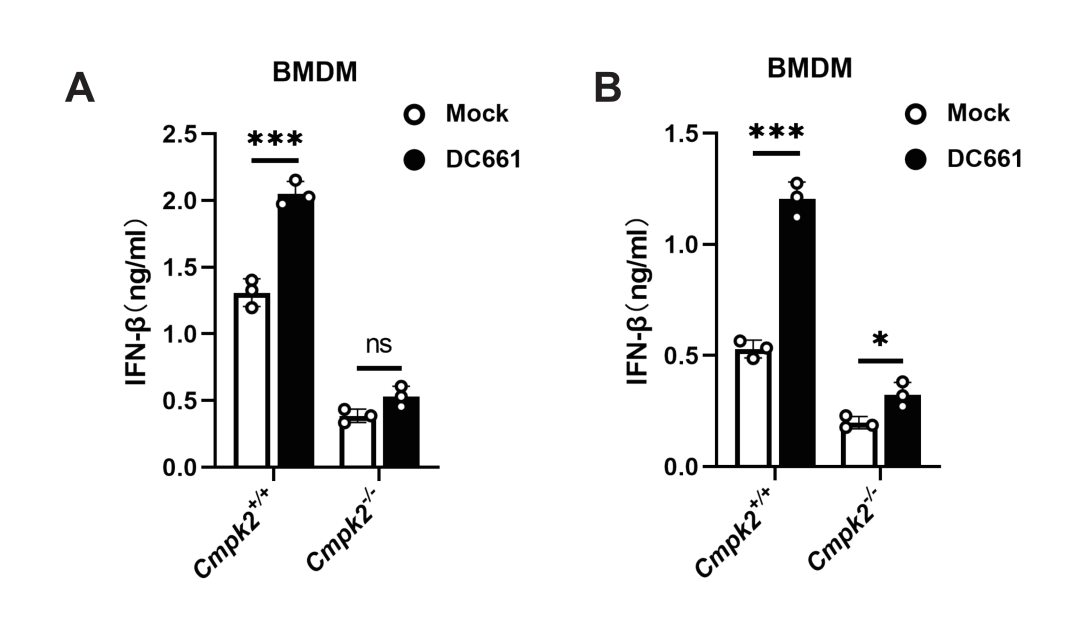


**Figure S6 DC661 enhances the antiviral response through targeting CMPK2.**

1. *Cmpk2^+/+^* or *Cmpk2^-/-^* BMDMs were pretreated with DMSO or DC661 and then infected with VSV for 12 h, after which IFN-β levels in the supernatants were detected by ELISA.
2. *Cmpk2^+/+^* or *Cmpk2^-/-^* BMDMs were pretreated with DMSO or DC661 and then infected with IAV for 12 h, after which IFN-β levels in the supernatants were detected by ELISA.

Statistical analysis was performed by one-way ANOVA (A and B). ns *P*>0.05, **P* < 0.05, ****P* < 0.001.

.

**Supplementary tables**

**Table S1. Primer sequences for qPCR**

#
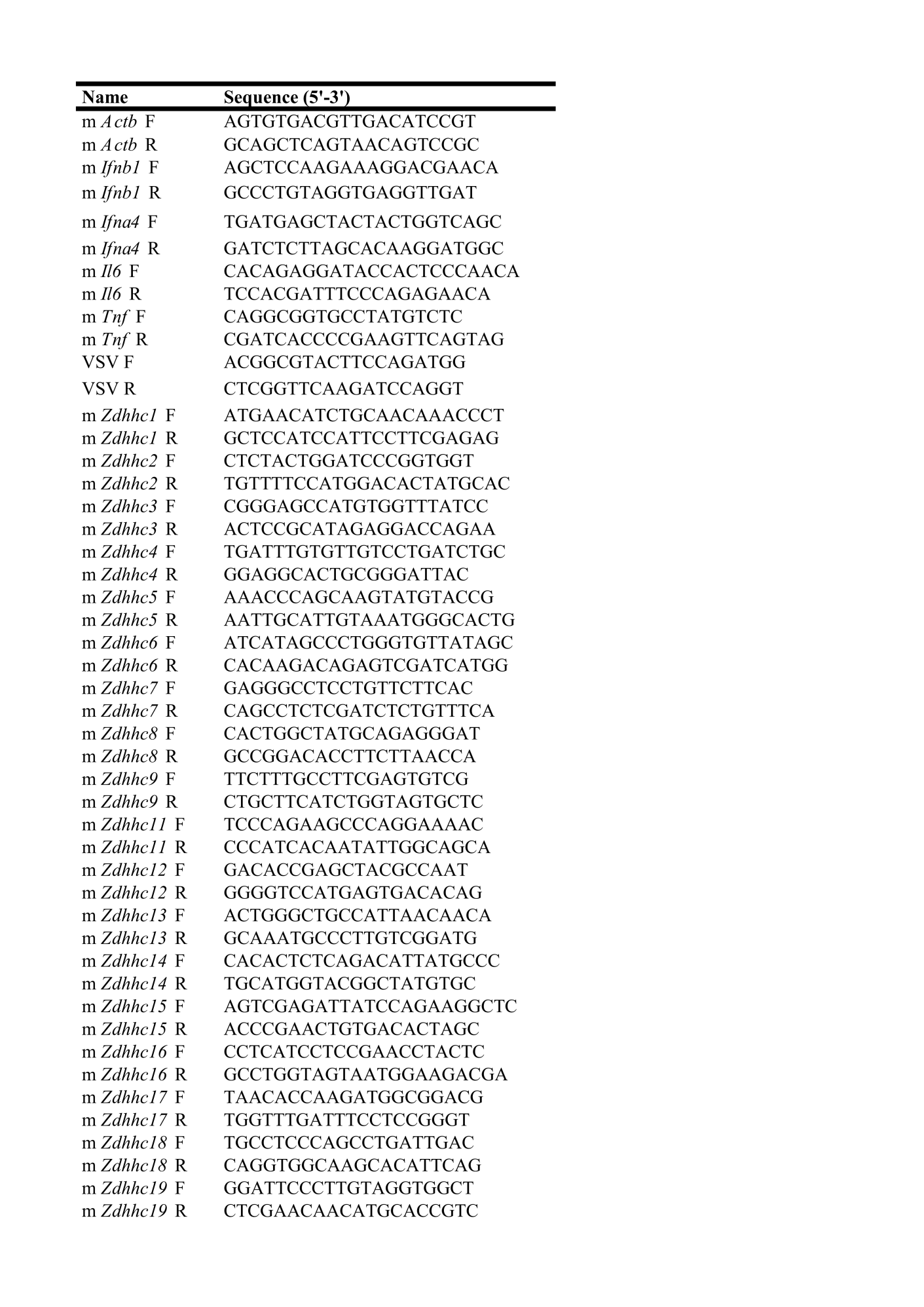


#
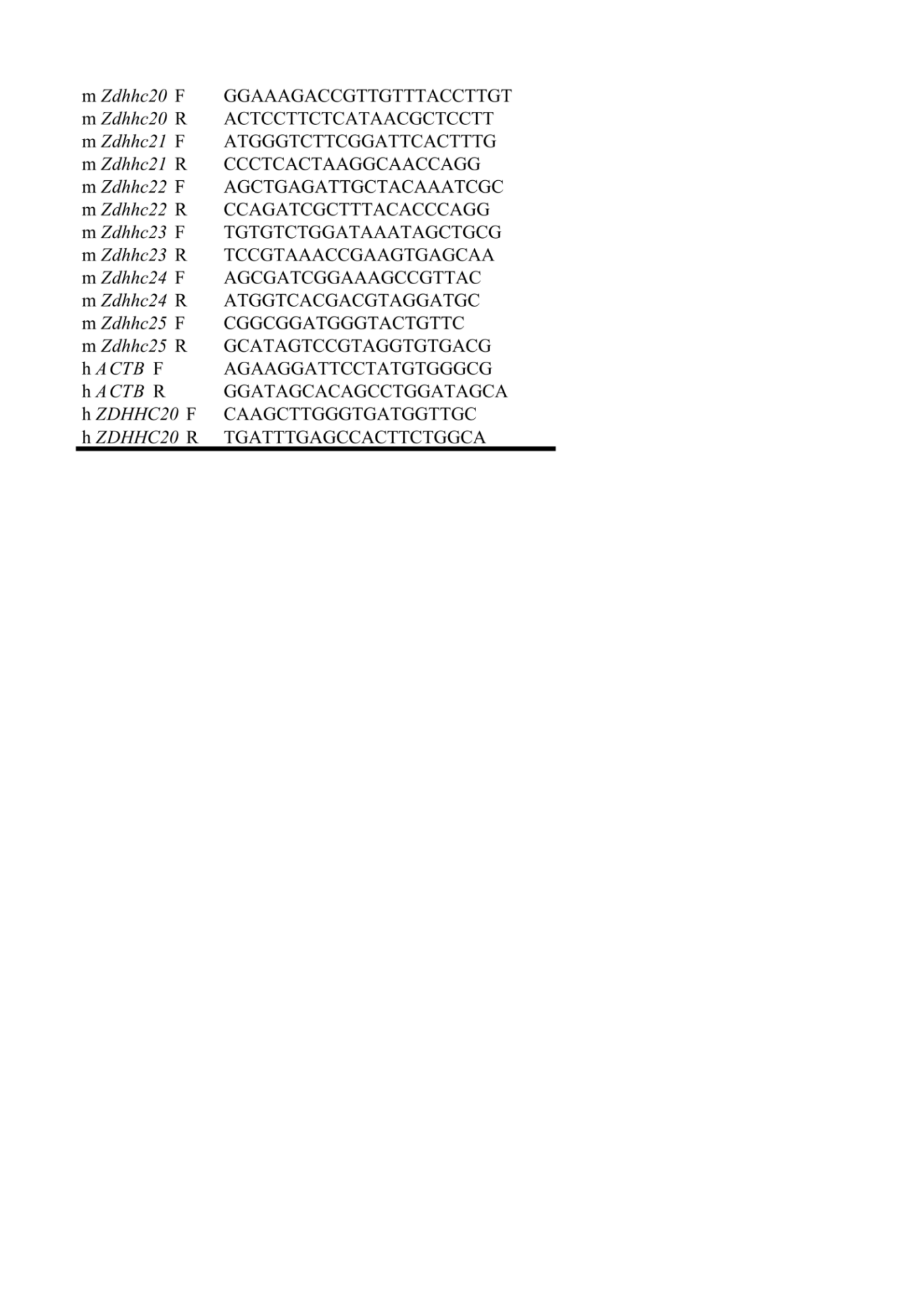


**Table S2. sgRNA and siRNA sequences**


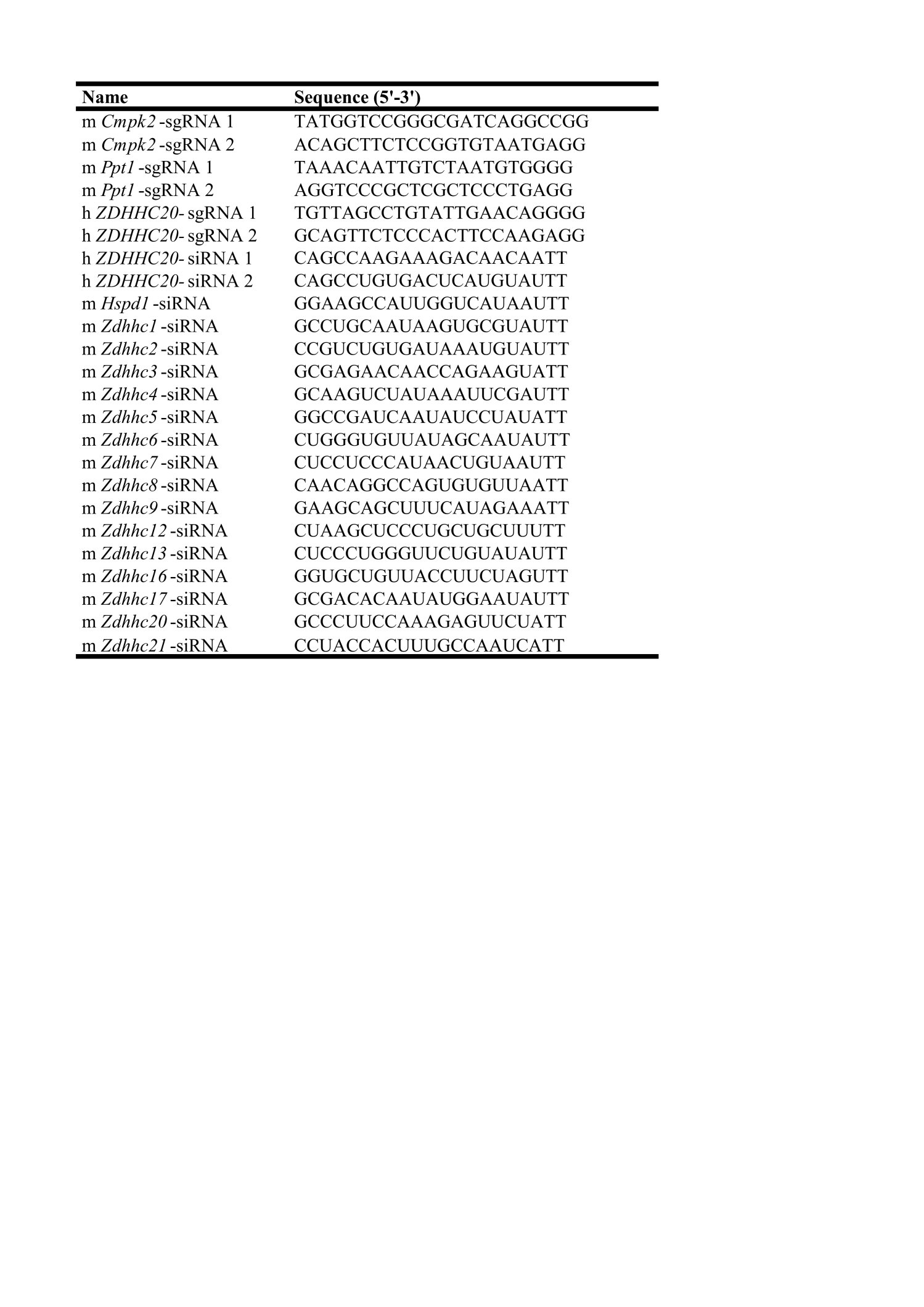

Supplement: Supplementary file 1 — Supporting File: advs75209‐sup‐0001‐SuppMat.docx. [file ADVS-9999-e75209-s001.docx]
